# Benchmarking on-the-Fly Machine Learning Force Fields for Ion Hydration: Structure, Coordination, and Exchange Dynamics across Monovalent and Divalent Cations

**DOI:** 10.1021/acs.jpcb.6c02837

**Published:** 2026-09-02

**Authors:** Mohammed S. Salha, Wuyang Lin, Gregory Chass, Devis Di Tommaso

**Affiliations:** Department of Chemistry, School of Physical and Chemical Sciences, 4617 Queen Mary University of London, Mile End Road, London E1 4NS, U.K.

## Abstract

Machine learning force fields (MLFFs) promise ab initio accuracy at a fraction of the computational cost of first-principles simulation, yet their fidelity for aqueous ion hydration across chemically diverse cation series remains systematically underexplored. Here, we benchmark an on-the-fly MLFF (OT-MLFF) framework, implemented in VASP via kernel-based Bayesian regression with adaptive DFT calls triggered when the estimated force error exceeds a set threshold, together with the pretrained, equivariant MACE foundation model applied off-the-shelf, against ab initio molecular dynamics (AIMD; CP2K, PBE-D3) and classical MD (CMD; Madrid-2019/TIP4P-2005) for aqueous solutions of Li^+^, Na^+^, K^+^, Cs^+^, Mg^2+^, and Ca^2+^. Across all six ions, the OT-MLFF reproduces a bulk water structure and the systematic increase of first-shell M–O distances down both cation series with near-AIMD accuracy, outperforming CMD. Coordination-number distributions reveal a mild OT-MLFF overcoordination of Li^+^ (modal CN 5 versus 4 in both AIMD and MACE) and Cs^+^, whereas the two divalent shells, including the previously problematic Ca^2+^, are reproduced by both machine-learned potentials, all three methods placing the Ca^2+^ coordination number at ≈6. MACE tracks AIMD most closely overall, its principal bias being a residual overcoordination of K^+^ and undercoordination of Cs^+^ in the diffuse monovalent shells. Angular distribution functions confirm accurate reproduction of the rigid octahedral geometry of Mg^2+^ but reveal systematic OT-MLFF smoothing of three-body features for more labile shells, including the O–Na–O shoulder near 65–70° and the O–Ca–O peak angle. The largest failures are kinetic: the OT-MLFF generates spurious first-shell water exchange for Mg^2+^, absent in AIMD, and completely suppresses the exchange of the dynamic Ca^2+^ shell, even though it reproduces the equilibrium coordination number of both ions, a clear dissociation between structural and dynamical fidelity. Velocity autocorrelation functions reveal a related failure, the OT-MLFF underestimating the cooperative cage confinement of the large monovalent ions Cs^+^ and K^+^. In each of these cases, the pretrained MACE model recovers the AIMD behavior that the on-the-fly potential misses. Collectively, these results establish that the OT-MLFF is robust for an equilibrium solvation structure but fails for three-body angular sensitivity and exchange kinetics in chemically distinct edge cases and that a deeper, higher-body-order, pretrained model such as MACE systematically narrows these gaps even without system-specific training. Targeted improvements such as augmented transition-state sampling, higher angular-resolution descriptors, and long-range electrostatic corrections are identified as high-priority directions for kinetic fidelity in next-generation electrolyte force fields.

## Graphical abstract

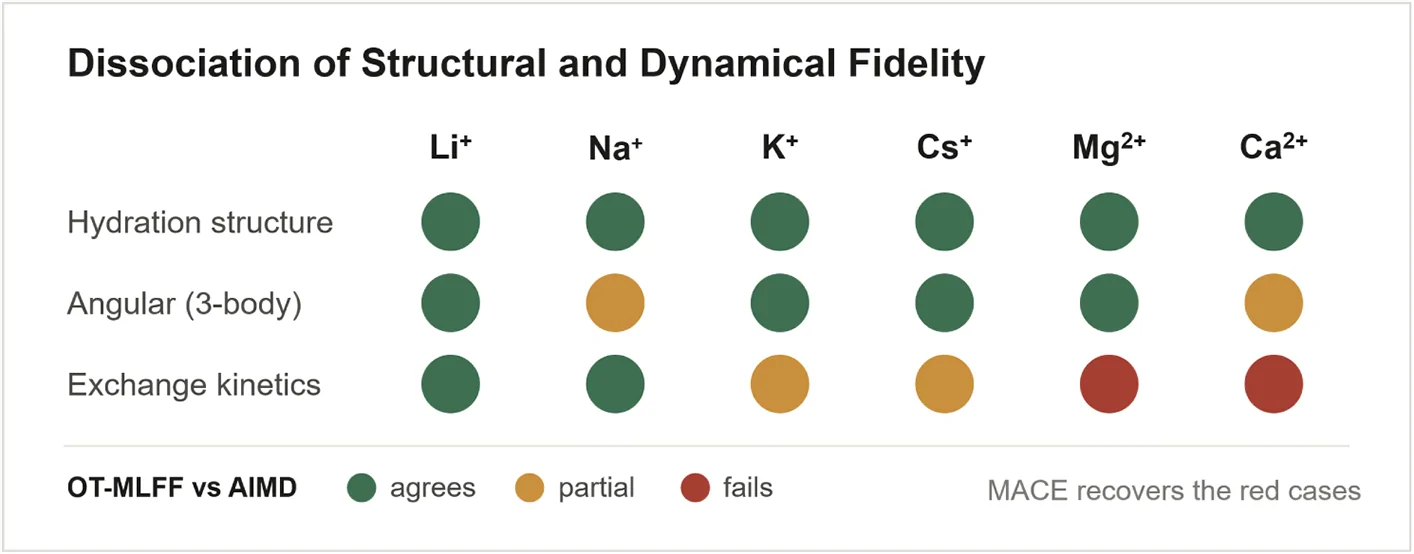

## 1 Introduction

Ion hydration is a central problem in computational chemistry, with direct relevance to electrochemistry, desalination, and the nucleation and growth of mineral precipitates.^1–3^ Classical molecular dynamics (CMD) simulations are computationally efficient and widely adopted to study ion hydration and electrolyte solutions, with simulation boxes containing hundreds to thousands of atoms and time scales routinely reaching the microsecond scale.^4^ However, classical force fields rely on fixed functional forms and empirical parametrization that cannot adapt to the local electronic environment of the ion, most critically misrepresenting the barriers governing water exchange in the first hydration shell of highly charged species. These limitations are most acute for fixed-charge, pairwise-additive models with integer ionic charges and simple Lennard-Jones interactions. More sophisticated force fields, such as Madrid-2019, partially address this through scaled ionic charges (0.85e monovalent, 1.7e divalent) that implicitly correct for mean-field polarization, yielding improved solution densities and activity coefficients across a wide concentration range.^5^ Nevertheless, this polarization correction is configuration-independent: the charge remains fixed regardless of instantaneous coordination geometry, and the model cannot respond explicitly to the local electric field gradient imposed by the hydration shell.^5,6^ For high-charge-density divalent ions, where ion-induced polarization of first-shell water is known to be large and anisotropic, this limitation is most consequential for coordination geometry and exchange barrier topology.^7,8^

Ab initio molecular dynamics (AIMD), typically based on density functional theory (DFT), computes interatomic forces quantum mechanically, inherently capturing electronic polarization and many-body effects that are inaccessible to fixed-charge classical models.^9^ This accuracy comes at a substantial computational cost: AIMD is generally limited to systems of fewer than a thousand atoms and time scales of tens to hundreds of picoseconds, restricting its applicability to dilute solutions and short-lived processes.^10^

These time-scale constraints become particularly consequential for the hydration dynamics of divalent ions, though through qualitatively different mechanisms depending on ionic radius.^11^ For Mg^2+^, whose rigid octahedral first hydration shell exchanges water on microsecond time scales, approximately 6 orders of magnitude slower than bulk water reorientation, standard AIMD cannot directly sample exchange events within a 50 ps simulation window, and free-energy methods such as metadynamics are required to characterize the exchange pathway and activation barrier.^12^ For Ca^2+^, the situation is distinct: with a first-shell mean residence time of approximately 60 ps from AIMD, exchange events are directly observable within standard simulation time scales, yet the larger ionic radius and flexible, nonoctahedral coordination geometry impose a different challenge: the accurate representation of a potential energy surface that supports both 6 and 7 coordination states separated by a low free-energy barrier.^13^

Machine learning force fields (MLFFs) address these limitations by training models on DFT-computed energies and forces, occupying an intermediate position in the accuracy–cost hierarchy: typically, two to four orders of magnitude cheaper than AIMD while substantially more expensive than CMD, they enable system sizes and time scales inaccessible to first-principles methods.^14^ Although more expensive than classical force fields by one to 3 orders of magnitude, this cost reduction relative to AIMD opens access to the time scales and enhanced sampling protocols required to characterize rare events such as water exchange in strongly hydrated ions.

Graph neural network (GNN) and equivariant message-passing architectures have rapidly become the dominant paradigm for MLFFs of aqueous systems, and a growing body of work has applied them specifically to ions in solution. Deep neural network potentials of the Deep Potential (DeePMD) family, trained on dispersion-corrected DFT, were among the first to reproduce the ion-specific “structure-making” and “structure-breaking” effects of dissolved salts: Avula, Klein, and Balasubramanian showed that DeePMD models capture the qualitatively opposite trends in water diffusivity for NaCl versus CsI solutions that conventional fixed-charge force fields fail to reproduce, tracing the difference to the distinct solvation-shell structure of Na^+^ and Cs^+^.^15^ Kernel-based Gaussian Approximation Potentials (GAP) have been applied to the same class of liquid-electrolyte problems and provide a useful efficiency and accuracy baseline against which message-passing models can be compared.^16^ Equivariant message-passing models, which encode higher-body-order correlations and directional information directly, have since improved on this class of description. A MACE-based many-body force field was recently shown to yield a quantitative improvement over DeePMD for the same anomalous-diffusion problem in NaCl and CsI solutions, attributable to a more faithful representation of the first-shell ion–water interaction,^17^ and systematic benchmarks of MACE model size and equivariance on aqueous NaCl have established that equivariant variants deliver both lower force errors and greater dynamical stability than their invariant counterparts.^18^ Beyond monovalent salts, equivariant potentials have been applied to more strongly hydrated cations: a MACE potential for aqueous MgCl_2_ reproduced the hydration structure, water-exchange kinetics, and transport properties of Mg^2+^, while simultaneously revealing that a purely local model underestimates the ion solvation free energy, a direct signature of the missing long-range electrostatics that local descriptors cannot capture.^19^ In parallel, pretrained “foundation” MLFFs, including the MACE-MP and Allegro families trained on broad materials data sets, now offer transferable potentials that can be deployed on aqueous systems off-the-shelf or with modest fine-tuning,^20,21^ although their accuracy for the condensed-phase, ion-specific observables of interest here remains under active evaluation.^22^ Collectively, these studies demonstrate that GNN- and transformer-class MLFFs can reach near-DFT fidelity for individual electrolyte systems, but they have largely focused on a small number of monovalent salts or a single divalent cation and on transport rather than coordination-shell structure and exchange kinetics. A systematic, multiobservable comparison across a chemically diverse cation series, the objective of the present work, has not yet been reported, and it is against this landscape that we position the autonomous on-the-fly Bayesian OT-MLFF alongside the pretrained equivariant MACE model.

A common feature of these approaches is that training data are assembled through a separate active learning cycle prior to deployment, requiring substantial data set curation before simulations can begin. The OT-MLFF framework implemented in VASP differs fundamentally: training data are generated autonomously *during* simulation via Bayesian force-error estimation, with DFT single-point calculations triggered adaptively whenever the predicted force uncertainty on any atom exceeds a set threshold.^23,24^ This integrated single-workflow approach substantially lowers the barrier to generating MLFFs for new chemical systems. Yet, despite demonstrated success for bulk solid-state and surface applications, its accuracy and failure modes for liquid-phase ion solvation remain uncharacterized: it is precisely this autonomous on-the-fly workflow, rather than the precured GNN potentials surveyed above, whose behavior across the diverse cation series we benchmark here.^26–28^

We apply this OT-MLFF framework to aqueous solutions of the monovalent alkali cations Li^+^, Na^+^, K^+^, and Cs^+^ and the divalent alkaline earth cations Mg^2+^ and Ca^2+^, selected to span the widest practical range of charge density, ionic radius, hydration shell rigidity, and ligand exchange time scale within a single, internally consistent computational framework. Within the monovalent series, ionic radii increase from 0.76 Å (Li^+^) to 1.67 Å (Cs^+^) and first-shell mean residence times from subpicosecond (Cs^+^, K^+^) to tens of picoseconds (Li^+^), providing a systematic test of OT-MLFF performance across compact, tightly bound shells and diffuse, highly labile ones.^29^ Mg^2+^ represents the extreme of rigid octahedral coordination with microsecond exchange kinetics; Ca^2+^, divalent but with a 40% larger ionic radius, occupies an intermediate regime of a flexible, nonoctahedral coordination number (CN) of approximately 7 and moderate exchange time scale, providing a stringent test of three-body angular sensitivity. Together, these six ions constitute a deliberate stress test of the OT-MLFF across the full dynamic range of ion–water interaction strength relevant to aqueous electrolyte modeling.

Structural and dynamical properties such as radial distribution functions, coordination-number distributions, angular distribution functions, water exchange rates and mean residence times, velocity autocorrelation functions, and metadynamics free-energy profiles are computed from OT-MLFF, AIMD, and CMD trajectories and compared systematically to establish where OT-MLFF succeeds, where it fails, and why.

## 2 Computational Details

Details of the solutions considered in this study, including the number of ions and water molecules, the cell lengths used, and the solution concentrations, are reported in Table S1 of the Electronic Supporting Information (ESI). Sixty-four water molecules were initially placed randomly in a cubic box (∼12 Å per side) using Packmol.^30^ For alkali metal chloride systems, two water molecules were randomly replaced with one cation and one anion; for alkaline earth metal chloride systems, three water molecules were replaced with one cation and two anions. For each system, we performed ab initio MD, MLFF MD, and classical MD simulations, as detailed below. The AIMD, OT-MLFF, and MACE simulations were in all cases performed at identical composition. As detailed in Table S1, AIMD cells contained one ionic formula unit in 58–62 water molecules (∼0.9 mol kg^–1^). The preparation protocol was identical: random packing with Packmol, classical NPT equilibration at 300 K and 1 atm to fix the cell volume, and identical propagation settings and ion count.

### 2.1 Ab Initio Molecular Dynamics

Our simulations were conducted using CP2K/Quickstep code, version 4.1.^31^ This software employs a hybrid Gaussian plane-wave implementation of DFT. For the exchange and correlation terms, we utilized the PBE generalized-gradient approximation.^32^ We incorporated the DFT-D3 dispersion correction developed by Grimme and coworkers to ensure an accurate description of the structure of liquid water.^33,34^ Core–valence interactions were handled using Goedecker–Teter–Hutter (GTH) pseudopotentials.^35^ Each atomic species was represented by a Goedecker-type double-ζ valence-polarized (DZVP-MOLOPT-SR-GTH) Gaussian basis set, optimized for use with GTH pseudopotentials in CP2K. We set the plane-wave kinetic energy cutoff (E_cut_) to 1000 Ry and restricted k-sampling to the Γ point of the Brillouin zone. Wave function optimization was performed with a tolerance of 10^–6^ au, and periodic boundary conditions were applied. A critical validation step involved comparing the solvation structure of our hydrated cations (Li^+^, Na^+^, K^+^, Cs^+^, Mg^2+^, Ca^2+^) with existing simulation and experimental measurements. AIMD simulations commenced from configurations equilibrated by CMD in the isothermal–isobaric (NPT) ensemble (T = 300 K, P = 1 atm), from which the equilibrium cell volume and atomic positions were extracted. The AIMD production runs were then performed in the canonical (NVT) ensemble at T = 300 K, with the simulation cell fixed at the volume determined by the preceding NPT CMD equilibration. This protocol is standard practice in AIMD simulations of aqueous systems: NPT equilibration with an empirical force field efficiently samples the correct liquid density at the target thermodynamic state point, after which the cell volume is held fixed for the DFT-based production run to avoid the prohibitive computational cost of constant-pressure AIMD and the well-known tendency of GGA functionals to overstructure liquid water and underestimate its density under NPT conditions, artifacts that are substantially mitigated by the inclusion of dispersion corrections (DFT-D3).^10,36–38^ A time step of 1.0 fs was used for all AIMD production runs. The structural and dynamical observables computed here (radial distribution functions, coordination numbers, angular distribution functions, and water exchange rates) are insensitive to the small residual density difference between the CMD-equilibrated and DFT-preferred volumes at these conditions, as confirmed by Wang et al. and Galib et al., who applied identical equilibration protocols for alkali-chloride and alkaline-earth aqueous systems.^36,37^ The NVT ensemble was maintained using a Nosé–Hoover chain thermostat with a relaxation time of 0.1 ps.

The AIMD trajectories serve as an independent PBE-D3 reference against which the machine-learned potentials are evaluated. We note that the AIMD (CP2K) and the OT-MLFF training calculations (VASP) share the same exchange–correlation functional and dispersion correction (PBE-D3) but differ in their core and basis representation: Gaussian and augmented plane wave with GTH pseudopotentials in CP2K versus pure plane waves with PAW in VASP. Because the functional is common to both, this representation difference is expected to affect absolute total energies and the intensity of the RDF peaks far more than the the structural observables of interest here, such as distances and coordination numbers. We have quantified it directly by repeating the AIMD simulation of aqueous NaCl in VASP at the same PBE-D3 level (Figure S4a). The two codes place the first Na–O maximum at effectively the same distance, but the VASP peak is appreciably more intense than the CP2K one, indicating that part of the apparent overstructuring of the OT-MLFF relative to the CP2K reference (Table 1) originates in the basis and pseudopotential representation rather than in the machine-learned potential itself. We therefore base our assessment on peak positions, coordination numbers, and trends across the ion series, which are robust to this difference, rather than on absolute peak intensities. We further confirm that our CP2K reference agrees with independent AIMD studies and experiment (Table S2).

**Table 1:** RDF Peak Positions and Heights for M–O in Each M–Cl System, Simulated with AIMD, MLFF, and CMD^a^

	AIMD		OT-MLFF		CMD		MACE	
Ion	r_max_	g_max_	r_max_	g_max_	r_max_	g_max_	r_max_	g_max_
**Li** ^**+**^	1.97	9.26	1.95	10.16	2.08	9.42	2.01	8.55
**Na** ^**+**^	2.39	5.13	2.34	6.43	2.48	5.95	2.41	5.38
**K** ^**+**^	2.85	3.48	2.79	4.23	2.65	5.55	2.75	3.60
**Cs** ^**+**^	3.25	2.45	3.05	3.65	3.17	2.08	3.13	2.26
**Mg** ^**2+**^	2.11	14.98	2.08	19.71	2.03	25.73	2.09	14.04
**Ca** ^**2+**^	2.39	10.70	2.34	11.01	2.45	9.77	2.37	9.76

a R_max = first peak position, g­(r_max) = peak height.

This comparison confirms that the chosen DFT level of theory reliably reproduces key structural features, including the position and amplitude of the first peak in the M–O RDF and the average coordination number within the first hydration shell. Notably, our results for Na^+^ and K^+^ are in close agreement with AIMD simulations using the strongly constrained and appropriately normed (SCAN) meta-GGA functional, which is recognized for its accuracy in modeling complex condensed-phase systems.^39^

### 2.2 On-the-Fly Machine Learning Force Field Molecular Dynamics

The “on-the-fly” technique implemented in VASP was used to generate MLFFs for six hydrated ion systems: Li^+^, Na^+^, K^+^, Cs^+^, Mg^2+^, and Ca^2+^.^23–25^ The chlorine (Cl^–^) ions were included as counterions to maintain charge neutrality. The projector augmented wave (PAW) method was used in combination with the PBE exchange-correlation functional and Grimme’s DFT-D3 dispersion correction to account for core–electron interactions, electronic exchange-correlation, and van der Waals forces, respectively.^40^ A plane-wave energy cutoff of 500 eV was applied, and the Brillouin zone was sampled at the Γ-point only, as is standard for disordered liquid-phase systems where the large simulation cell and absence of long-range periodicity render *k*-point sampling beyond the zone center unnecessary. The threshold for invoking DFT calculations to refine the MLFF, based on the maximum Bayesian-estimated error of force (BEEF), was set to 0.04 eV/Å. This threshold was selected following the protocol of Jinnouchi et al.^25^ This value governs the balance between training efficiency and force field accuracy: when the predicted force uncertainty on any atom exceeds this value, a full DFT single-point calculation is triggered, the resulting energies and forces are added to the training set, and the MLFF is immediately updated.^24^ Over the course of the MLFF generation which comprised four sequential 50 ps runs per system with progressively reduced BEEF thresholds, the following DFT calculations were triggered for each system: 1266 for LiCl, 1603 for NaCl, 1260 for KCl, 1051 for CsCl, 1319 for MgCl_2_, and 1266 for CaCl_2_. The threshold for max BEEF gradually reduced: 0.2, 0.1, 0.08, and 0.04 eV Å^–1^. This confirms that the on-the-fly protocol generates a compact but representative training data set without manual curation. Local energy contributions were computed using a radial cutoff of 10 Å and an angular cutoff of 6 Å. After the generation of the MLFF, each system was subjected to a 50 ps MLFF-based MD simulation in the NPT ensemble at 300 K and 1 bar, employing the Langevin thermostat (friction coefficient γ = 10 ps^–1^, applied uniformly to all atomic species) with a time step of 1 fs. The following hyperparameters were used uniformly across all six systems: radial cutoff radius 10.0 Å; angular cutoff radius 6.0 Å, encompassing up to the third nearest-neighbor shell; 12 radial basis functions; 8 angular basis functions; angular resolution *l*_max_ = 3 (up to g-type angular functions); regularization parameter λ = 10^–5^ to prevent overfitting; Bayesian noise parameters σ_E_ = 0.01 eV (energy) and σ_F_ = 0.01 eV/Å (forces).

As a validation of the OT-MLFF equation of state, a separate 50 ps NPT simulation of pure liquid water (64 molecules, 300 K, 1 bar) yielded a density of 0.998 g cm^–3^, in excellent agreement with the experimental value of 0.997 g cm^–3^ at 298 K and confirming that the OT-MLFF accurately reproduces the thermodynamic state point of liquid water prior to introduction of ions.

The accuracy of the resulting force fields was further assessed out-of-sample. For each electrolyte, 200 statistically decorrelated frames were extracted from the final 90% of the production trajectory, propagated in pure prediction mode (ML_MODE = run), so that no DFT is computed on them during the run, and recomputed with single-point PBE-D3 calculations at the training level. The OT-MLFF reproduces the reference forces with an RMSE of 78–84 meV Å^–1^ (mean 81 meV Å^–1^) and a force-component correlation R = 0.994 for every system and the total energies to within 1.6–2.0 meV atom^–1^. The accuracy is essentially uniform across the ion series, the RMSE varying by less than 8% between the most and least accurate system, so the ion-specific structural and kinetic discrepancies reported in Section 3 do not arise from a locally poorer force fit for ions. Full details, per-system error statistics, and force and energy parity plots are given in Section S3, Table S3 and Figure S3. As a further check, the CaCl2 trajectory was extended to 430 ps: the Ca–O radial distribution function from the extended run is indistinguishable from that of the 50 ps production run (Figure S4b). All results reported in the main text are taken from the 50 ps production trajectories.

### 2.3 MACE Foundation Model Molecular Dynamics

Machine learning MD was additionally performed with the MACE-MP-0 foundation model (medium variant), E(3)-equivariant message-passing neural network with higher-body-order messages (correlation order 3), and a built-in short-range repulsive baseline.^20^ In contrast to the on-the-fly OT-MLFF, MACE-MP-0 is pretrained on the MPTrj data set (≈1.5 million PBE structures from the Materials Project) and was applied off-the-shelf. We deliberately applied MACE-MP-0 in a zero-shot manner, without fine-tuning on system-specific DFT data. This is the more stringent transferability test: a foundation model that reproduces the target observables with no system-specific training demonstrates genuine generalization, whereas fine-tuning on the same aqueous-ion configurations used for evaluation would conflate transferability with in-domain interpolation. As shown below, the untuned model already recovers the two discrepancies of principal concern, the Li^+^ coordination number and the Ca^2+^ exchange kinetics, so fine-tuning was not required to address them and would be expected only to refine an already-accurate description. Simulations were run in LAMMPS using the MACE pair style with GPU (Kokkos/CUDA) acceleration, in the NVT ensemble at 300 K maintained with a Nosé–Hoover chain thermostat (temperature-damping constant 0.1 ps, matching the AIMD thermostat coupling time, Section 2.1), with a 1 fs time step for 50 ps, starting from the same equilibrated 1 M configurations and simulation cells as the AIMD and OT-MLFF runs. As the MACE-MP-0 training set is PBE (without an explicit dispersion correction), its potential energy surface corresponds to a slightly different reference level from the PBE-D3 used for AIMD and the OT-MLFF, which is borne in mind when comparing peak intensities.

### 2.4 Classical Molecular Dynamics

CMD simulations were performed using the pairwise Lennard-Jones (LJ) potential model developed by Zeron et al., commonly referred to as the Madrid-2019 force field.^5^ This model describes aqueous solutions of Li^+^, Na^+^, K^+^, Mg^2+^, Ca^2+^, and Cl^–^, employing the TIP4*P*/2005 water model.^41^ In Madrid-2019, monovalent and divalent ions are represented using scaled charges of 0.85 and 1.7 electron units, respectively. This approach enables highly accurate predictions of solution densities, even at elevated concentrations.^5^ Since the Madrid-2019 force field does not include parameters for Cs^+^, equilibrated CsCl solutions were generated using the LJ potential model from the AMBER force field database.^42^ For each system, 6 ns of CMD simulations were carried out in the NPT ensemble to equilibrate the simulation cell volume. Classical MD simulations were propagated with the leapfrog integrator and a 1 fs time step, with all bonds constrained using the LINCS algorithm. The equations of motion were integrated in the NPT ensemble at 300 K and 1 bar. The temperature was maintained using the velocity-rescale (stochastic velocity rescaling) thermostat of Bussi et al.^42,43^ with a coupling time of 0.4 ps, and the pressure was controlled with the Parrinello–Rahman barostat (isotropic coupling, coupling time 2.0 ps, reference pressure 1.0 bar, water compressibility 4.5 × 10^–5^ bar^–1^). Electrostatics were treated with the particle-mesh Ewald (PME) method, and short-range electrostatic and van der Waals interactions used a 0.6 nm cutoff. The final configuration from each CMD run was then used as the starting point for subsequent AIMD simulations.^44^

### 2.5 Metadynamics Simulations

Well-tempered metadynamics simulations were performed using LAMMPS coupled with PLUMED 2.9.4, with energies and forces evaluated at each step.^45,46^ The simulation was performed using periodic boundary conditions as the classical MD, meaning an NVT ensemble at 300 K using a Nosé–Hoover thermostat with a coupling time of 0.1 ps and a time step of 1 fs. The collective variable (CV) was the Mg–O coordination number, computed with a rational switching function (PLUMED RATIONAL) with parameters R_0_ = 1.1 Å (0.11 nm), D_0_ = 1.9 Å (0.19 nm), *n* = 4, *m* = 8, and a cutoff of 3.5 Å (0.35 nm), following the parametrization of Raiteri et al. that we have previously validated for hydrated Mg^2+^.^47,48^ Well-tempered Gaussian hills of height 1.0 kJ/mol and width σ = 0.1 in the CN were deposited every 500 steps (0.5 ps) with a bias factor of 5 at a well-tempered temperature of 300 K. Lower and upper restraining walls were placed at CV = 2.5 and 7.5 with force constants of 5000 and 2000 kJ/mol, respectively, to prevent sampling of unphysical coordination states. The CV and bias potential were recorded every 10 steps. The simulation was conducted in six consecutive 5 ns segments for a total production time of 30 ns, with restart files written every 5 ns to allow safe recovery and resumption between segments. This was repeated using the AMBER force field and a simple Lennard-Jones potential with ε = 0.1 kcal/mol, σ = 2.5 Å, and a cutoff of 10.0 Å. This generic parametrization was used solely to benchmark the structural pipeline and software setup and is not intended to represent a physically validated force field for ion hydration. The PLUMED input was kept consistent, controlling the definition of the CV as the number of oxygen atoms coordinated to the Mg center. The deposition of the Gaussian hills and their height/width remained consistent with the methodology described above, except for a lack of upper and lower restraints for both the LJ and AMBER methods. The classical (AMBER and Lennard-Jones) and MACE free-energy profiles were computed in LAMMPS with PLUMED, whereas the OT-MLFF profile was obtained by coupling the VASP on-the-fly MLFF to PLUMED through the native VASP–PLUMED interface; all engines used the identical collective variable and well-tempered metadynamics parameters, so the profiles are directly comparable.

## 3 Results and Discussion

### 3.1 Structural Properties

#### 3.1.1 Radial Distribution Function

Structural characteristics of aqueous electrolyte solutions containing alkali metal ions (M = Li^+^, Na^+^, K^+^, Cs^+^) and alkaline-earth metal ions (M = Mg^2+^ and Ca^2+^), as simulated using AIMD, MLFF MD, and CMD, can be inferred from the water–water radial distribution functions (RDFs) for O–O and O–H pairs (Figure 1) and the ion–water RDFs for M–O pairs (Figure 2). The radial distribution function (RDF) g*_αβ_* (r) describes how atomic density varies as a function of distance from a reference atom, providing a measure of the probability of finding an atom of type *α* at a distance *r* from an atom of type *β* relative to an ideal random distribution. RDFs are a fundamental tool for characterizing the local structure of liquids and solutions, as they reveal key features such as coordination shells, hydrogen-bonding patterns, and solvation environments. In the context of aqueous electrolyte solutions, water–water RDFs (O–O and O–H) provide insights into the perturbation of the hydrogen-bond network by dissolved ions, while ion–water RDFs (M–O) quantify the strength and geometry of ion hydration.

**Figure 1:**
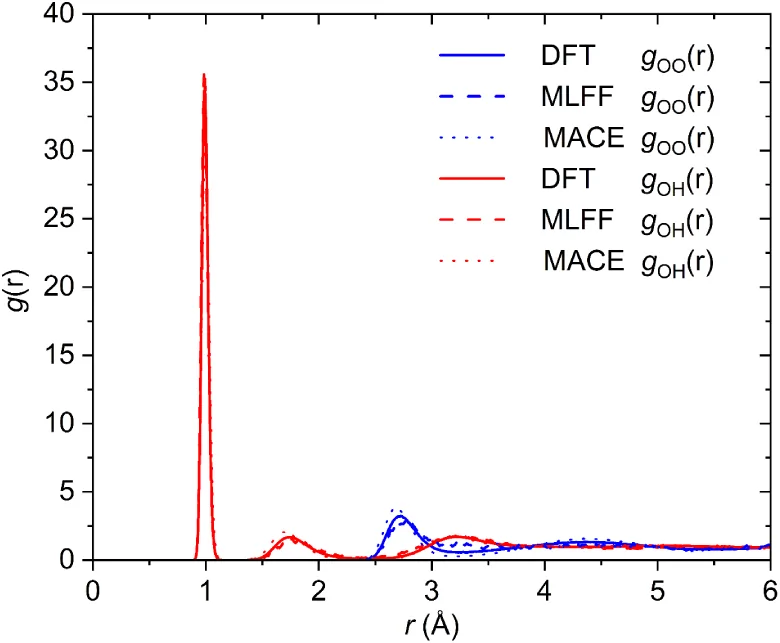
Oxygen–oxygen (O–O) and oxygen–hydrogen (O–H) radial distribution function, g­(r), for aqueous NaCl solution computed using AIMD (solid) and OT-MLFF MD (dashed) and MACE (dotted).

**Figure 2:**
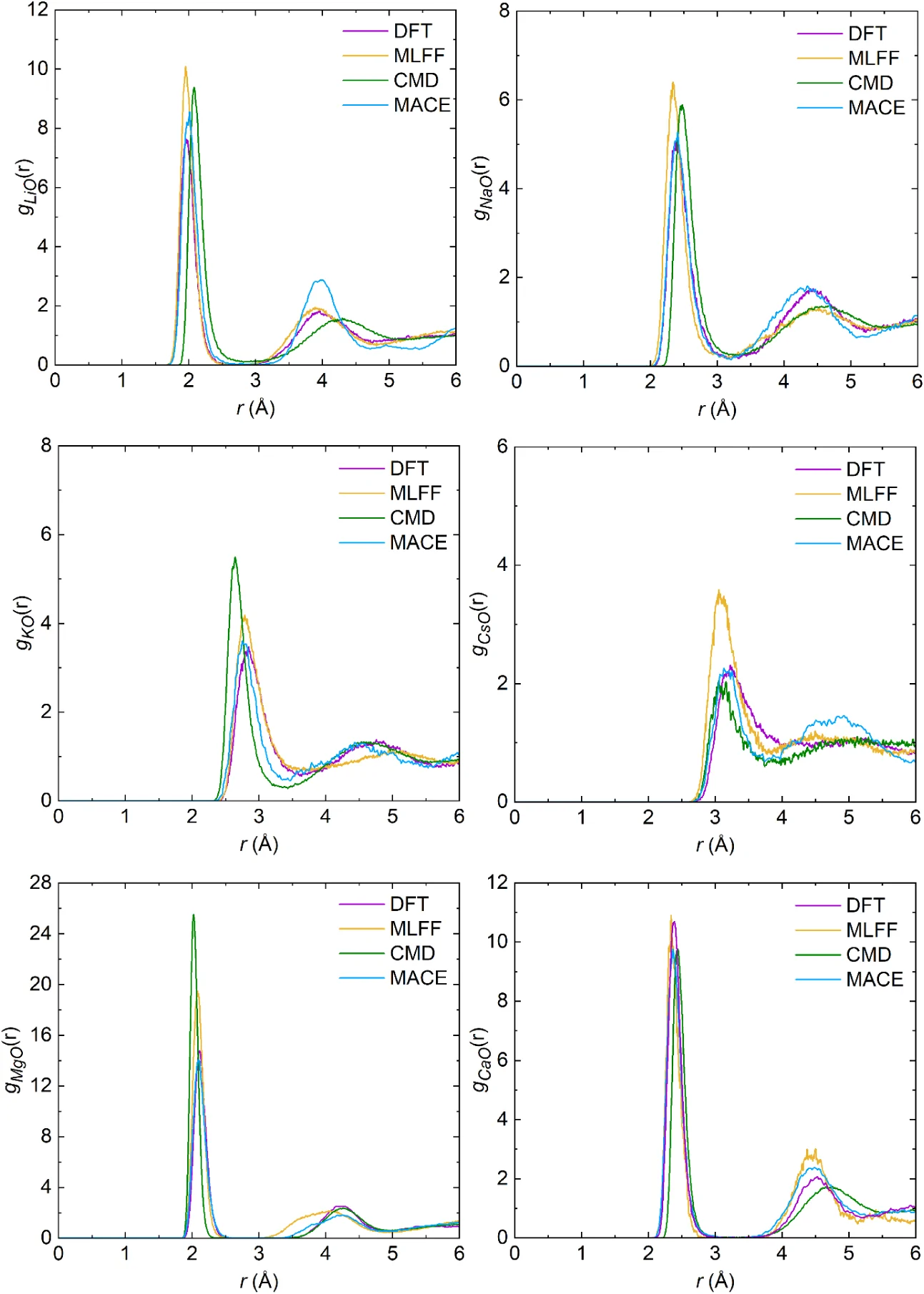
Comparison of the radial distribution functions, g­(r), for metal–oxygen pairs in various electrolyte systems: (a) Li–O, (b) Na–O, (c) K–O, (d) Cs–O, (e) Mg–O, and (f) Ca–O. Simulations were performed using ab initio molecular dynamics (AIMD, solid purple), the on-the-fly machine learning force field (OT-MLFF, solid yellow), the MACE foundation model (MACE, solid light blue), and the classical Madrid-2019 force field (CMD, solid green).

Figure 1 establishes the water–water baseline for aqueous NaCl. The OT-MLFF reproduces the AIMD O–H and O–O RDFs quantitatively in both peak position and amplitude, the intramolecular O–H peak at ∼1.0 Å, and the first- and second-neighbor O–O peaks at ∼2.8 and ∼3.2 Å that encode the hydrogen-bond network, with the curves overlapping beyond the first shell and residual large-*r* deviations within the statistical noise of the 50 ps AIMD trajectory. The MACE profiles are shown alongside. This close agreement confirms that the OT-MLFF captures the water structure at the 10 Å cutoff and provides a sound baseline for the ion-specific M–O comparisons of Figure 2.

The ion–water RDFs in Figure 2 reveal that the average metal–oxygen distance in the first hydration shell increases systematically down both the alkali and alkaline earth series. Small, highly charged ions such as Li^+^, Mg^2+^, and Ca^2+^ (and to a lesser extent Na^+^) form tightly bound, well-defined first solvation shells, as indicated by sharp, high-intensity RDF peaks. In contrast, larger, low-charge-density ions like K^+^ and Cs^+^ exhibit broader, lower-intensity peaks, reflecting more diffuse and labile hydration. This trend persists into the second hydration shell, where the broader features for K^+^ and Cs^+^ indicate weaker correlations with more distant water molecules. The MLFF reproduces these structural trends with high fidelity, particularly for rigid, well-defined shells, accurately capturing peak positions, intensities, and widths. The MLFF consistently reproduces the first-shell peak positions and intensities across the alkali and alkaline earth series, demonstrating superior agreement with the DFT reference compared to the classical model, particularly in capturing the subtle coordination environments of larger cations and divalent species. By comparison, CMD tends to overestimate RDF peak heights for Li^+^ and Na^+^ and underestimate them for Mg^2+^, Ca^2+^, and K^+^. For example, the first minimum of the RDF of Li–O (around 2.5–3.0 Å), the MLFF matches the “zero” probability region of the DFT reference, whereas the classical model shows a significant “leak”. These observations underscore the role of ion charge density in shaping solvation: small, highly charged ions form compact, stable hydration shells, whereas larger, low-charge-density ions form diffuse, dynamic shells that are inherently more challenging to model. Capturing these distinctions is essential for predicting ion transport, solvation energetics, and coordination-dependent reactivity in aqueous environments. MACE reproduces the M–O peak positions closely for Li^+^, Na^+^, Mg^2+^, and Ca^2+^ (within 0.02–0.04 Å of the AIMD values in Table 1), with somewhat larger inward shifts for K^+^ and Cs^+^ (∼0.1 Å), and predicts divalent peak intensities marginally below the AIMD reference.

#### 3.1.2 Ion–Water Coordination Shell Distributions

Coordination-number (CN) distributions, computed by integrating the M–O RDF up to its first minimum, quantify the probability of finding a given number of water molecules in the first hydration shell of each cation and provide a direct measure of how accurately each simulation method captures ion-specific hydration structures (Figure 3). The trends across the cation series reflect the well-established dependence of first-shell occupancy on ionic radius and charge density: small, highly charged ions form compact, tightly bound shells with low and sharply defined CNs, while large, weakly polarizing ions accommodate more water molecules in diffuse, fluctuating shells with broad CN distributions.

**Figure 3:**
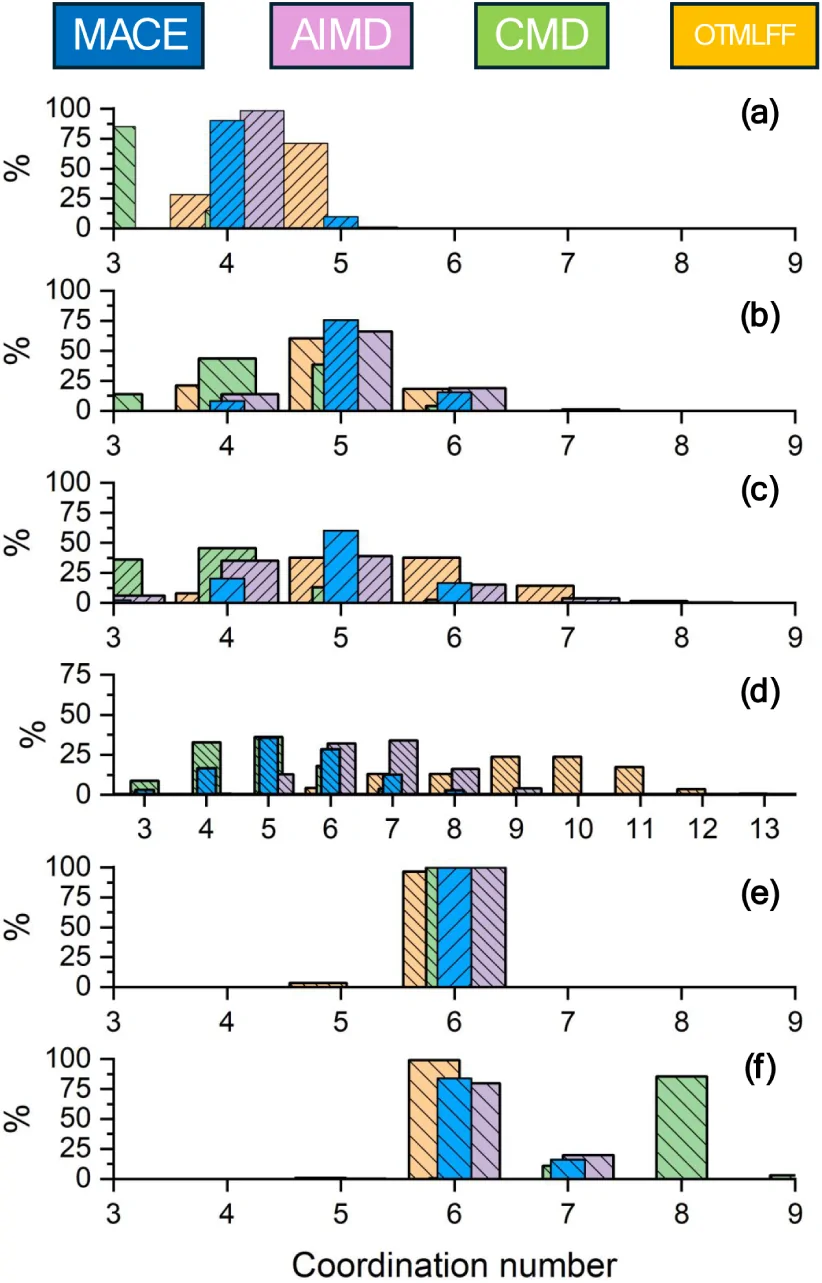
Distribution of the ion–water coordination number in aqueous solutions of (a) LiCl, (b) NaCl, (c) KCl, (d) CsCl, (e) MgCl_2_, and (f) CaCl_2_. The distributions were calculated using the ion-specific first-shell cutoff radii listed in Table 2, taken from the first minimum of the AIMD metal–oxygen radial distribution function and applied identically to all four methods. Results are compared for AIMD, OT-MLFF, MACE, and classical (CMD) molecular dynamics simulations.

**Li** ^**+**^: AIMD predicts an essentially singular hydration environment at CN = 4, reflecting the compact first shell imposed by the small ionic radius and high charge density of Li^+^. MACE reproduces this first shell almost exactly (mean CN = 4.1), recovering the steep short-range repulsion that governs Li^+^ hydration. CMD is qualitatively consistent, with a modal CN of 3–4. The OT-MLFF, however, predicts a modal CN of 5, overcoordinating Li^+^ by approximately one water relative to both AIMD and MACE. The most plausible interpretation of this overcoordination is that the OT-MLFF does not fully reproduce the steep short-range repulsive wall of the compact Li+ shell; we cannot, however, exclude a contribution arisingfrom a less accurate description of the balance between ion–water and water–water interactions at this small ionic radius. The observation that MACE, whose architecture includes a built-in short-range repulsive term, recovers the AIMD coordination number almost exactly is consistent with, though not a unique proof of, a short-range-repulsion origin. Li^+^ remains the most challenging monovalent case for the OT-MLFF and the clearest case in which the pretrained, many-body MACE model outperforms the on-the-fly kernel description.

**Na** ^**+**^: The more labile hydration environment of Na^+^ is reflected consistently across all three methods. AIMD yields a broad distribution centered at CN = 5, with significant populations at CN = 4 and CN = 6, indicative of frequent first-shell fluctuations. The OT-MLFF reproduces this distribution closely, capturing both the modal CN and the width of the distribution, the best agreement observed among the monovalent series. CMD shifts the distribution toward lower coordination, with a pronounced population at CN = 4 and reduced weight at CN = 6, underestimating first-shell occupancy relative to AIMD. MACE reproduces the AIMD Na^+^ distribution equally well (mean CN ≈5.0), matching both the modal coordination number and the width of the distribution.

**K** ^**+**^: Method-dependent differences become more pronounced for K^+^. AIMD predicts a broad distribution spanning CN = 4–7, with maximum probability at CN = 5–6, consistent with the diffuse and weakly structured hydration shell expected for this larger alkali ion. The OT-MLFF reproduces the breadth of this distribution but shifts slightly toward higher coordination numbers, increasing the populations at CN = 6 and CN = 7. CMD deviates substantially, strongly favoring CN = 3–4 with negligible population above CN = 6, indicating a severe underestimation of the effective K^+^–water interaction strength in the Madrid-2019 force field at this ionic size. MACE shifts the K^+^ distribution higher still, overcoordinating to a mean CN of ≈6.2 against the AIMD mean of ≈4.8, the larger of the two MACE deviations for this ion.

**Cs** ^**+**^: Cs^+^ has the largest and most diffuse hydration shell of the monovalent series. AIMD predicts a broad distribution with a mean CN of ≈6.7, reflecting the large ionic radius and weak electrostatic field of Cs^+^. The two machine-learned potentials bracket this value: the OT-MLFF overcoordinates Cs^+^ (mean CN ≈9.2), whereas MACE undercoordinates it (mean CN ≈5.4), the largest MACE discrepancy of the series. CMD fails severely, predicting a distribution centered at CN = 4–5 with negligible population above CN = 6, an underestimation relative to AIMD attributable to the use of the Amber force field parameters for Cs^+^ (Madrid-2019 does not include Cs^+^), which substantially underestimate the Cs^+^–water interaction range. This methodological asymmetry should be borne in mind when interpreting CMD results for this ion specifically.

**Mg** ^**2+**^: The CN distribution for Mg^2+^ is described with excellent consistency across all three methods. AIMD predicts an essentially singular occupation at CN = 6, reflecting the well-known rigid octahedral [Mg­(H_2_O)_6_]^2+^ complex and its microsecond-scale ligand-exchange kinetics. Both the OT-MLFF and CMD reproduce this result quantitatively, making Mg^2+^ the most robustly described ion across all methods and confirming that the highly constrained, electronically stiff coordination environment of Mg^2+^ is amenable to description at all three levels of theory. MACE likewise reproduces the rigid six-coordinate octahedron exactly (CN = 6), in quantitative agreement with AIMD, the OT-MLFF, and CMD and confirming that Mg^2+^ is robustly described at every level of theory.

**Ca** ^**2+**^: In contrast to the pronounced method dependence seen at higher concentrations, the 1 M Ca^2+^ coordination-number distributions are in good mutual agreement. AIMD predicts a mean CN of 6.2, a dominant CN = 6 population with a substantial secondary CN = 7 population, consistent with the established flexibility of the Ca^2+^ first shell and its tendency toward higher coordination than Mg^2+^. Both the OT-MLFF (mean CN ≈6.0) and MACE (mean CN ≈6.2) reproduce this behavior, capturing the dominant six-coordinate shell and, for MACE, the residual seven-coordinate flexibility. CMD overcoordinates Ca^2+^ in the opposite direction, placing the distribution at CN = 8 with minor populations at CN = 7 and CN = 9. The near-quantitative agreement of both machine-learned potentials with AIMD indicates that the six/seven-coordinate Ca^2+^ shell is well-described at the OT-MLFF and MACE levels and is not, at this concentration, a failure mode of the on-the-fly approach.

Taken together, the CN distributions reveal method-specific biases that are largest for the classical model. AIMD provides the most physically consistent reference across the full ion series. MACE tracks AIMD most closely overall, matching Li^+^ (CN 4), Na^+^ (5), Mg^2+^ (6), and Ca^2+^ (≈6.2), though it overcoordinates K^+^ and undercoordinates Cs^+^, indicating a residual bias for the large, weakly structured monovalent shells. The OT-MLFF reproduces Na^+^, Mg^2+^, and Ca^2+^ with near-AIMD accuracy and substantially outperforms CMD for the large monovalent cations, its principal remaining biases being a mild overcoordination of Li^+^ and Cs^+^. CMD systematically undercoordinates large monovalent cations, severely so for Cs^+^, and overcoordinates Ca^2+^, while performing well only for Mg^2+^. Overall, both MLFFs describe the divalent shells well, with the remaining discrepancies confined to the diffuse monovalent shells of K^+^ and Cs^+^.

#### 3.1.3 Angular Distribution Functions

The O–M–O angular distribution functions (ADFs, Figure 4) probe three-body coordination geometry, with peak width measuring shell rigidity: sharp peaks denote a well-defined geometry, whereas broad profiles indicate a labile one. Across the series, the ADFs track the rigid-to-diffuse trend already evident in the RDFs and coordination numbers. The high-charge-density ions Mg^2+^ and Li^+^ (and, less sharply, Ca^2+^ and Na^+^) show well-resolved peaks, and the O–Mg–O distribution peaks sharply at θ ≈90°, consistent with the octahedral [Mg­(H_2_O)_6_]^2+^ geometry, whereas the large, weakly polarizing K^+^ and Cs^+^ give broad, near-featureless profiles spanning ∼50–120°; the near-uniform O–Cs–O spread indicates no preferred coordination angle, consistent with the high, variable CN of this ion (Section 3.1.2).

**Figure 4:**
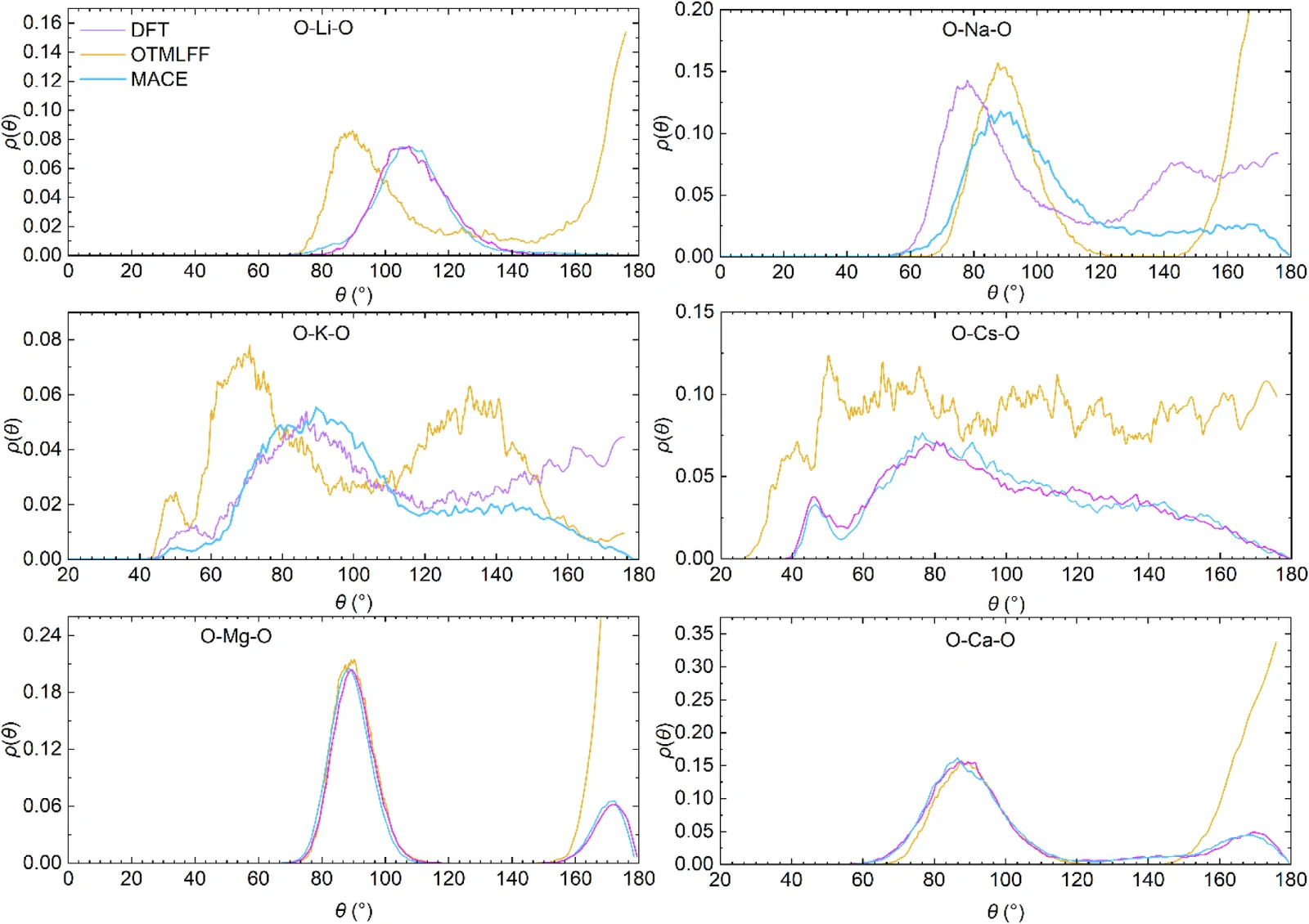
Comparison of the angular distribution functions, g­(r), for oxygen–metal–oxygen triplets in various electrolyte systems: (a) O–Li–O, (b) O–Na–O, (c) O–K–O, (d) O–Cs–O, (e) O–Mg–O, and (f) O–Ca–O. Simulations were performed using ab initio molecular dynamics (DFT, purple), on-the-fly machine learning force field (OT-MLFF, yellow), and MACE (light blue).

The ability of the OT-MLFF to replicate the AIMD ADFs is ion-dependent and reveals specific limitations in capturing three-body angular correlations. The OT-MLFF accurately reproduces the Mg^2+^ ADF, correctly predicting both the peak position at θ ≈90° and its narrow width, reflecting the fact that the rigid octahedral shell presents an unambiguous and well-sampled energy minimum for the model to learn. For the more labile systems, however, the OT-MLFF introduces systematic artifacts of varying character. For Na^+^, the shoulder near θ = 65–70° present in the AIMD ADF, indicative of a minor but physically meaningful subpopulation of undercoordinated configurations, is absent in the OT-MLFF, which instead produces a smoother, unimodal distribution. For Li^+^, analogous cusps and shoulders in the AIMD profile are similarly suppressed.

The Ca^2+^ case warrants particular attention. The OT-MLFF overestimates the O–Ca–O peak angle relative to AIMD, imposing a more octahedral angular geometry on an ion whose AIMD coordination is nonoctahedral. Importantly, at the 1 M concentration examined here, this angular stiffening is no longer accompanied by a coordination-number error; the OT-MLFF, MACE, and AIMD all report a Ca^2+^ CN of ≈6 (Section 3.1.2), so the distortion manifests instead in the kinetics: we interpret this over-rigid angular landscape as suppressing the coordination-number fluctuations (CN 6 ⇌ 7) through which Ca^2+^ exchanges water; this interpretation is consistent with the complete suppression of Ca^2+^ exchange in the OT-MLFF and with its recovery by the higher-angular-resolution MACE model, although the angular and kinetic observations remain correlative rather than causally isolated. The angular error is therefore best understood as a signature of an overstiff three-body potential energy surface for Ca^2+^ rather than as a coordination-counting artifact. The MACE O–M–O angular distributions are included in Figure 4 for comparison.

Notably, and somewhat surprisingly, the OT-MLFF largely reproduces the broad, noisy pattern of the Cs^+^ ADF despite failing to capture its caging dynamics in the VACF analysis (Section 3.3). This apparent inconsistency reflects the distinction between the equilibrium angular structure, which the OT-MLFF captures adequately at the pair-distribution level, and dynamical cage effects, which depend on the curvature and barriers of the potential energy surface rather than its average topology.

The inconsistency of OT-MLFF errors across the cation series reflects the inherent difficulty of representing three-body interactions relative to the two-body pair correlations described by the RDFs. The angular component of the potential energy surface is highly nonlinear and varies qualitatively across the ion series, from the steep, well-defined well of Mg^2+^ to the flat, barrier-free landscape of Cs^+^. In highly disordered systems, the energy landscape contains many nearly degenerate states separated by small gradients, producing noisy, inconsistent forces in the AIMD training data; as an interpolation scheme trained on these forces, the OT-MLFF defaults to a smoother approximation that suppresses the cusps and shoulders defining short-range angular order and generalizes less reliably to poorly sampled configurations. We associate this with the locality of the kernel descriptor, whose finite angular cutoff and resolution are examined further in Section 4. We emphasize that our data establish the presence and ion dependence of these angular errors but do not isolate a single cause; the support from the MACE comparison is suggestive rather than definitive.

### 3.2 Dynamics of the Ionic Solvation Shell

Table 2 summarizes the coordination numbers (CN), water exchange event counts (N_ex_), normalized exchange rates (N_ex_ per 10 ps), and mean residence times (MRT) for the first and second hydration shells of each metal chloride solution, derived from the AIMD, OT-MLFF, and MACE trajectories. MRTs were computed as MRT = (t_sim_ × CN)/N_ex_, where N_ex_ is the number of exchange events counted over the trajectory of length t_sim_ subject to the dwell-time criterion defined below. Together, these quantities characterize the kinetic stability of each hydration shell and provide a dynamical complement to the equilibrium structural analysis of Sections 3.1.1–3.1.3 Throughout, a first-shell exchange is counted only when a water molecule crosses the coordination-shell boundary and remains beyond it for longer than a minimum dwell time of t* > 0.5 ps; this criterion removes transient recrossings, i.e., subpicosecond rattling of a molecule across the boundary that does not constitute a genuine ligand-exchange event.^49^ Because true exchanges are separated from such recrossings by a clear time scale gap, the residence times are insensitive to the precise value of t* over the physically reasonable range (∼0.2–2 ps), and the categorical differences between methods discussed below are unaffected by this choice.

**Table 2:** Number of Accounted Water Exchange Events $\left(N_{\mathrm{ex}}^{H_{2}O}\right)$ in the First and Second Coordination Shells of the Metal Ions Li^+^, Na^+^, K^+^, Cs^+^, Mg^2+^, and Ca^2+^, Values of $\left(N_{\mathrm{ex}}^{H_{2}O}\right)$ Normalized to the Number of Cations in Solution^a^

			First shell				Second shell			
System	b (m)	t_sim_ (ps)	CN	N_ex_	MRT (ps)	r_cat_ (Å)	CN	N_ex_	MRT (ps)	r_cat_ (Å)
LiCl-AIMD	0.9	50	4.0	7 ± 3	28.6 ± 10.8	2.65	17.7	1331 ± 36	0.66 ± 0.02	4.90
LiCl-MLFF	0.9	50	5.0	32 ± 6	7.8 ± 1.4		19.6	1828 ± 43	0.54 ± 0.01	
LiCl-MACE	0.9	50	4.1	19 ± 4	10.8 ± 2.5		17.9	1493 ± 39	0.60 ± 0.02	
NaCl-AIMD	0.9	50	5.1	178 ± 13	1.4 ± 0.1	3.15	21.4	2022 ± 45	0.53 ± 0.01	5.35
NaCl-MLFF	0.9	50	5.0	144 ± 12	1.7 ± 0.1		17.7	1028 ± 32	0.86 ± 0.03	
NaCl-MACE	0.9	50	5.0	143 ± 12	1.7 ± 0.1		21.4	1611 ± 40	0.66 ± 0.02	
KCl-AIMD	0.9	50	4.8	536 ± 23	0.4 ± 0.02	3.50	21.9	1852 ± 43	0.59 ± 0.01	5.55
KCl-MLFF	0.9	50	5.6	638 ± 25	0.4 ± 0.02		23.3	956 ± 31	1.22 ± 0.04	
KCl-MACE	0.9	50	6.2	479 ± 22	0.6 ± 0.03		23.9	1987 ± 45	0.60 ± 0.01	
CsCl-AIMD	0.9	50	6.7	809 ± 28	0.4 ± 0.01	3.90	28.4	1976 ± 44	0.72 ± 0.02	6.00
CsCl-MLFF	0.9	50	9.2	849 ± 29	0.5 ± 0.02		25.0	635 ± 25	1.97 ± 0.08	
CsCl-MACE	0.9	50	5.4	687 ± 26	0.4 ± 0.02		27.5	1565 ± 40	0.88 ± 0.02	
MgCl_2_-AIMD	0.9	50	6.0	0	>50^b^	2.85	19.2	1365 ± 37	0.70 ± 0.02	5.00
MgCl_2_-MLFF	0.9	50	6.0	14 ± 4	21.4 ± 5.7		21.3	2876 ± 54	0.37 ± 0.01	
MgCl_2_-MACE	0.9	50	6.0	0	>50^b^		17.6	1148 ± 34	0.77 ± 0.02	
CaCl_2_-AIMD	0.9	50	6.2	10 ± 3	31.0 ± 9.8	3.20	19.6	1446 ± 38	0.68 ± 0.02	5.40
CaCl_2_-MLFF	0.9	50	6.0	0	>50^b^		22.7	1449 ± 38	0.78 ± 0.02	
CaCl_2_-MACE	0.9	50	6.2	7 ± 3	44.3 ± 16.7		24.1	1660 ± 41	0.73 ± 0.02	

a Mean residence time (MRT) of water molecules in the first hydration shell was computed using the relation MRT = (t_sim_ × CN)/N_ex_, where t_sim_ is the period of simulation (in ps) and CN is the average ion–water coordination number of the first and second coordination shell, which was obtained from the integration of the ion–water radial distribution function up to the ion-specific first-shell cutoff radii (r_cut_) taken from the first minimum of the AIMD metal–oxygen radial distribution function and applied identically to all four methods. Concentration (b) in mol kg^–1^. MRT in ps. Uncertainties are Poisson counting errors on N_ex_ (±N_ex_^1/2^) propagated to the MRT (±MRT/N_ex_^1/2^); rows with N_ex_ = 0 are quoted as lower bounds (>50 ps). The small divalent first-shell counts carry correspondingly larger relative errors, but the qualitative distinctions, spurious Mg^2+^ first-shell exchange and suppressed Ca^2+^ exchange in the OT-MLFF, exceed these uncertainties.

b ^*a*^ *“>50” denotes no exchange event observed within t*_*sim*_ *(rigid first shell, e.g., Mg* ^*2+*^ *), for which the residence time exceeds the simulation length.*

#### 3.2.1 General Trends

Across all three methods, the kinetic stability of the first hydration shell decreases systematically with increasing ionic radius and decreasing charge density, consistent with the well-established dependence of ligand exchange rates on ion–water interaction strength. Li^+^ and Mg^2+^ exhibit the lowest exchange frequencies and longest first-shell MRTs, reflecting their compact, tightly bound coordination environments. At the other extreme, K^+^ and Cs^+^ show high exchange rates and short MRTs, consistent with their diffuse, weakly structured first shells and the broad CN distributions identified in Section 3.1.2. The second hydration shell is consistently more labile than the first across all systems, with higher CNs and shorter MRTs, reflecting the progressive transition toward bulk-like water behavior with increasing distance from the ion.

**Li^+^**: AIMD yields a first-shell MRT of 28.6 ps with an exchange rate of 1.4 events per 10 ps, reflecting a compact but not fully inert hydration shell. The OT-MLFF predicts a substantially higher exchange rate of 6.4 events per 10 ps and a corresponding reduced MRT of 7.8 ps. MACE lies between the two (MRT = 10.8 ps, 3.8 events per 10 ps), markedly closer to AIMD. This acceleration of exchange in the OT-MLFF is linked to its mild CN overestimation (Section 3.1.2): a modal CN of 5 rather than 4 implies a less sterically constrained first shell. MACE, which recovers the AIMD coordination number, correspondingly recovers a more physical exchange rate.

**Na** ^**+**^: AIMD yields a first-shell MRT of 1.4 ps and an exchange rate of 35.6 events per 10 ps, reflecting the well-known lability of the Na^+^-hydration shell. The OT-MLFF reproduces these values closely, predicting an MRT of 1.7 ps and an exchange rate of 28.8 events per 10 ps, the best quantitative agreement in the monovalent series, consistent with the accurate CN distribution reported in Section 3.1.2. MACE performs equally well, with a first-shell MRT of 1.7 ps (28.6 events per 10 ps), matching both the OT-MLFF and the AIMD.

**K** ^**+**^: AIMD predicts a first-shell MRT of 0.4 ps and an exchange rate of 107.2 events per 10 ps, indicating a highly labile shell with a near-continuous exchange. The OT-MLFF reproduces both the MRT (0.4 ps) and the exchange rate (127.6 events per 10 ps) with good fidelity, consistent with the near-AIMD CN distribution for this ion. The modest overestimation of the exchange rate in the OT-MLFF is commensurate with its slight overestimation of the CN for K^+^. MACE likewise reproduces the highly labile K^+^ shell (MRT = 0.6 ps, 95.8 events per 10 ps).

**Cs** ^**+**^: AIMD yields a short first-shell MRT (0.4 ps) and a high exchange rate (161.8 events per 10 ps), consistent with the large, weakly polarizing Cs^+^ shell. The OT-MLFF reproduces the exchange rate reasonably well (169.8 events per 10 ps) and the first-shell MRT (0.5 ps); MACE is similar (MRT of 0.4 ps, 137.4 events per 10 ps). The second-shell MRT is notably longer in the OT-MLFF (1.97 ps vs 0.72 ps in AIMD), suggesting that the OT-MLFF overstabilizes the second coordination shell of Cs^+^, consistent with the softer effective cage inferred from the VACF analysis (Section 3.3). MACE gives an intermediate second-shell MRT (0.88 ps) closer to AIMD.

**Mg** ^**2+**^: No first-shell exchange events are observed in the AIMD trajectory over 50 ps, consistent with the well-established microsecond-scale water exchange kinetics of Mg^2+^ and confirming that the 50 ps AIMD window is far too short to sample this process. The OT-MLFF predicts 14 exchange events over the same period, corresponding to an exchange rate of 2.8 events per 10 ps and a first-shell MRT of 21.4 ps. This is a physically significant discrepancy: the OT-MLFF substantially underestimates the kinetic barrier to first-shell water exchange in Mg^2+^, producing exchange events on a time scale that is orders of magnitude faster than experiment and AIMD. This finding is consistent with the metadynamics free-energy analysis in Section 3.4, which shows that the OT-MLFF systematically flattens the free-energy barriers between coordination states for Mg^2+^. Although the OT-MLFF correctly reproduces the equilibrium CN = 6 and the rigid octahedral ADF for Mg^2+^, the barrier topology of the potential energy surface, which governs exchange kinetics rather than the equilibrium structure, is insufficiently steep. This represents the most consequential kinetic failure of the OT-MLFFs identified in this study. MACE, by contrast, reproduces the AIMD result exactly, registering no first-shell exchange over 50 ps and thus correctly preserving the kinetic inertness of the Mg^2+^ octahedron; the spurious Mg^2+^ exchange is a failure mode of the OT-MLFF that the pretrained model avoids. In contrast to a recently reported bespoke MACE potential trained specifically for aqueous MgCl_2_, which reproduces Mg^2+^ exchange kinetics,^19^ we find that the *autonomous on-the-fly* OT-MLFF generates spurious first-shell Mg^2+^ exchange absent in AIMD, whereas the off-the-shelf MACE-MP-0 foundation model preserves the correct kinetic inertness, isolating the failure to the on-the-fly kernel workflow rather than to the MACE architecture per se.

**Ca** ^**2+**^: AIMD predicts 10 first-shell exchange events over 50 ps, with an MRT of 31 ps, reflecting a moderately stable but genuinely dynamic first shell. The OT-MLFF predicts zero exchange events, yielding an effectively infinite first-shell MRT. MACE, in contrast, recovers the dynamic character of the shell, registering 7 exchange events (MRT 44 ps) in good agreement with AIMD. This is the clearest case in which the two machine-learned potentials diverge kinetically despite agreeing structurally: all three methods place the Ca^2+^ coordination number at ≈6 (Section 3.1.2), yet the OT-MLFF renders the shell kinetically rigid, suppressing the CN 6 ⇌ 7 fluctuations through which exchange proceeds, whereas MACE preserves them. The OT-MLFF failure for Ca^2+^ is therefore dynamical rather than structural, as it reproduces the average coordination but not the softness of the potential energy surface that permits ligand exchange.

#### 3.2.2 Second-Shell Dynamics

Across all three methods, second-shell CNs are substantially higher than first-shell values and MRTs are consistently shorter, confirming the progressive transition to bulk-like water behavior. MACE reproduces the AIMD second-shell MRTs closely for every ion, the largest deviation being 0.16 ps for Cs^+^. The OT-MLFF is less consistent: it markedly overestimates the second-shell MRT of the large monovalent cations, most notably Cs^+^ (1.97 vs 0.72 ps) and K^+^ (1.22 vs 0.59 ps), while underestimating it for Mg^2+^ (0.37 vs 0.70 ps). The overestimation for Cs^+^ and K^+^ suggests a tendency to overstabilize the outer shell of weakly bound, diffuse ion-water structures, and may reflect the finite radial cutoff of the OT-MLFF descriptor (10 Å), which captures second-shell interactions but cannot account for the cooperative many-body effects that govern the rapid turnover of outer-shell water molecules, which is consistent with the Cs^+^ and K^+^ caging failures identified in Section 3.3.

Taken together, the exchange dynamics show that the OT-MLFF reproduces first-shell kinetics well for Na^+^ and K^+^ but diverges from AIMD for Li^+^ (overestimated exchange rate), Mg^2+^ (spurious exchange events absent in AIMD), and Ca^2+^ (complete suppression of exchange). MACE improves on all three: it recovers the AIMD Li^+^ rate more closely, preserves the kinetic inertness of Mg^2+^ (zero exchange), and reproduces the dynamic Ca^2+^ shell. The remaining OT-MLFF kinetic errors, traceable for Li^+^ to a residual structural bias but for Mg^2+^ and Ca^2+^ to an over-rigid potential energy surface, despite a correct equilibrium structure, are largely absent from the pretrained MACE description.

### 3.3 Caging Effect in the Hydration Shell of Ions

The corresponding MACE velocity autocorrelation functions are shown alongside in Figure 5. The velocity autocorrelation function (VACF) describes the time evolution of the velocity correlation of an ion with its initial value and provides a sensitive probe of the dynamic cage imposed by the surrounding hydration shell. A deep, sustained negative minimum (backscattering) indicates strong confinement, where the motion of the ion is rapidly reversed by collisions with the cage walls. A shallow or rapidly recovering minimum indicates a soft, easily deformable cage. The normalized VACFs for all six cations, computed from AIMD, OT-MLFF, and MACE trajectories, are presented in Figure 5.

**Figure 5:**
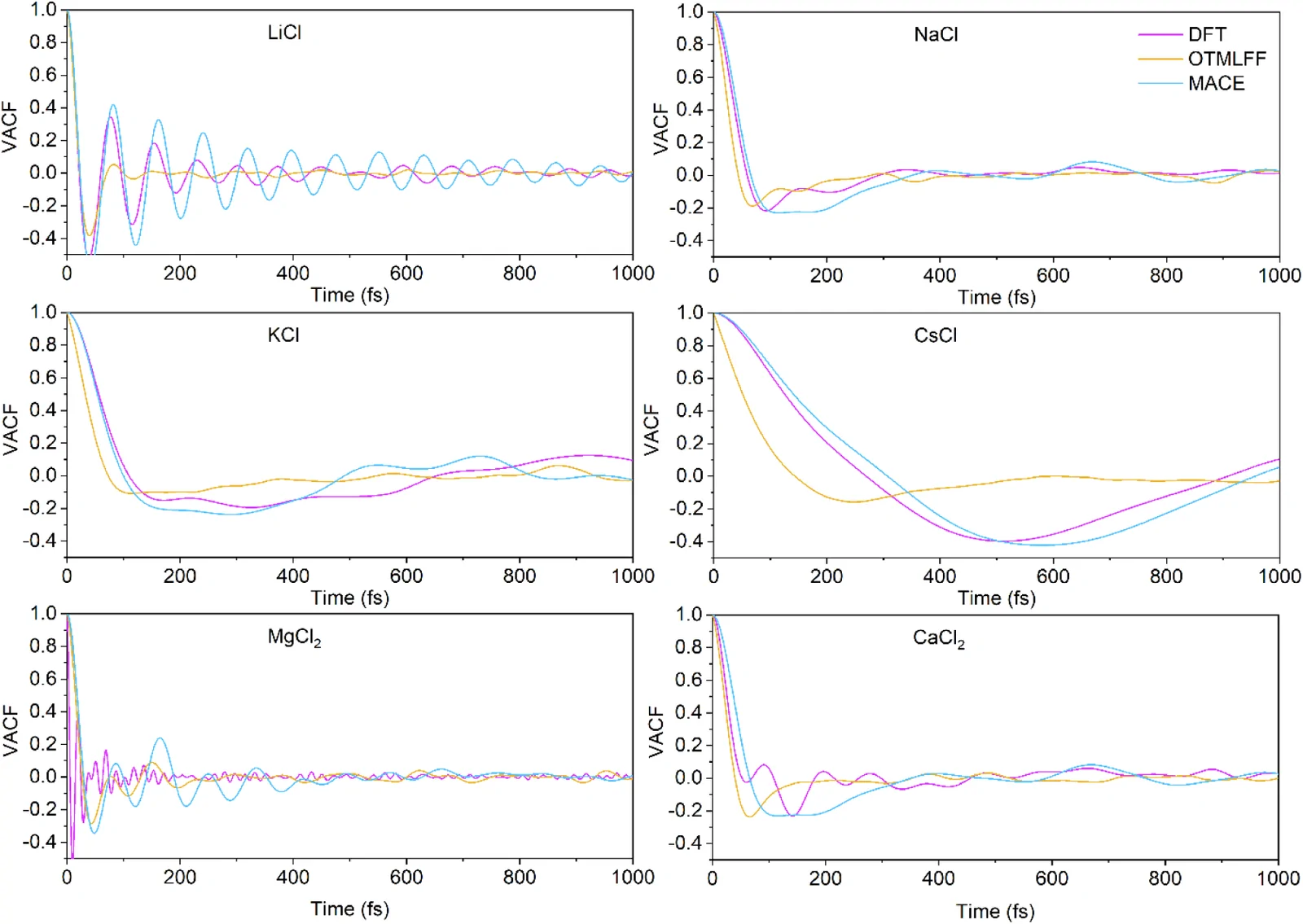
Normalized velocity autocorrelation functions (VACFs), Cv­(t) = ⟨v­(t)·v(0)⟩/⟨v(0)·v(0)⟩, of the metal cations in aqueous MCl*_n_* solutions computed from AIMD, OT-MLFF, and MACE trajectories: (a) Li^+^, (b) Na^+^, (c) K^+^, (d) Cs^+^, (e) Mg^2+^, and (f) Ca^2+^. The depth and timing of the backscattering minimum reflect the rigidity of the first hydration shell cage: deeper, earlier minima indicate stronger confinement. High-frequency oscillations superimposed on the initial decay in (e) and (f) are characteristic of the rattling motion of divalent cations within their stiff, electrostatically rigid coordination shells.

#### 3.3.1 Monovalent Series

The AIMD VACFs reveal a systematic dependence of caging behavior on ionic radius and mass across the monovalent series. Li^+^ exhibits the deepest and earliest backscattering minimum (∼−0.4 at ∼20 fs), consistent with strong cage confinement imposed by its compact, tightly bound first hydration shell, a direct dynamical signature of the short Li^+^–O distance and high first-shell rigidity established in Sections 3.1.1 and 3.1.2. Na^+^ and K^+^ display progressively shallower minima at later times, reflecting their larger ionic radii, weaker ion–water interactions, and more labile first shells. The OT-MLFF reproduces this systematic progression faithfully for Li^+^, Na^+^, and K^+^, correctly capturing both the depth and timing of the backscattering minimum for each ion. This agreement is consistent with the accurate equilibrium CN distributions and exchange dynamics reported for Na^+^ and K^+^ in Sections 3.1.2 and 3.2 and confirms that the OT-MLFF potential energy surface is sufficiently accurate to reproduce short-time cage dynamics for these ions even where longer-time scale exchange kinetics show some deviation.

The most significant VACF discrepancy in the monovalent series, and indeed across the entire data set, is observed for Cs^+^. The AIMD VACF decays to a deep negative minimum of approximately −0.4 at ∼500 fs, indicating strong and long-lived cage confinement despite the large ionic radius and weak charge density of Cs^+^. This counterintuitive result reflects the collective hydrodynamic response of the large, high-CN hydration shell: although individual Cs^+^–water interactions are weak, the sheer number of coordinating water molecules and their cooperative dynamics impose a substantial effective cage. The OT-MLFF fails to reproduce this behavior: the predicted VACF decays far more slowly, reaches only a shallow near-zero minimum, and recovers to positive values well before the AIMD curve reaches its minimum. This represents a qualitative, not merely quantitative, failure: the OT-MLFF fundamentally misrepresents the caging mechanism for Cs^+^. A similar but less pronounced discrepancy is observed for K^+^, where the OT-MLFF again underestimates the depth of the backscattering minimum relative to AIMD. We note that the OT-MLFF production runs were propagated with a Langevin thermostat of friction coefficient γ = 10 ps^–1^ (Section 2.2), corresponding to a thermostat relaxation time of 1/γ = 100 fs; as the Cs^+^ backscattering minimum occurs at ∼500 fs, five thermostat relaxation times later, direct momentum damping by the thermostat cannot be excluded as a contributing factor and this should be borne in mind when comparing VACFs computed under different thermostatting schemes.

#### 3.3.2 Divalent Series

The divalent systems present a qualitatively different VACF profile. The AIMD VACFs for both Mg^2+^ and Ca^2+^ display prominent high-frequency oscillations superimposed on the initial backscattering decay, most pronounced for Mg^2+^ given the exceptional rigidity of its octahedral first hydration shell. These oscillations reflect the rattling motion of the ion within a stiff, well-defined cage, a dynamical signature of the strong, directional Mg^2+^–water electrostatic interaction. In contrast to the monovalent failures described above, the OT-MLFF reproduces the VACF profiles of both Mg^2+^ and Ca^2+^ with good fidelity, correctly capturing the frequency, amplitude, and damping of the high-frequency oscillations. While the OT-MLFF correctly reproduces the equilibrium Ca^2+^ CN distribution (Section 3.1.2), it fails to reproduce the exchange dynamics (Section 3.2). Notably, despite this kinetic failure, the short-time cage dynamics of Ca^2+^, which are governed by the curvature of the ion–water potential at the equilibrium geometry rather than by the relative stability of different coordination states, are accurately described. For Mg^2+^, the agreement extends across the full 1000 fs window, consistent with the rigidity and long residence times that make the Mg^2+^ cage insensitive to the coordination exchange failures identified in Section 3.2.

The AIMD VACFs, particularly for the divalent systems, exhibit appreciably higher statistical noise than the OT-MLFF curves. Both trajectories span the same 50 ps production window (Section 2.2); the difference in apparent noise instead reflects the inherently noisier force estimates from the ab initio potential energy surface relative to the smoother, interpolated OT-MLFF force field. The pattern of OT-MLFF successes and failures across the VACF analysis is consistent with the structural and kinetic findings of Sections 3.1 and 3.2 and points to a coherent underlying limitation. The OT-MLFF accurately describes cage dynamics when the caging mechanism is governed by strong, short-range, directional ion–water interactions, as for Li^+^, Mg^2+^, and Ca^2+^ at short times. However, it underestimates cage confinement when it arises from the collective, cooperative response of a large, weakly bound hydration shell, as for Cs^+^ and K^+^. This distinction maps directly onto the locality approximation inherent to the kernel-based OT-MLFF descriptor: strong, localized ion–water interactions are well-represented within the radial and angular cutoffs, while the cooperative many-body dynamics of large, diffuse hydration shells require longer-range correlations that the finite cutoff cannot fully capture. The contrast with MACE is instructive and reflects a difference of architecture rather than of tuning. The OT-MLFF is a kernel-based Bayesian-regression potential built on fixed two- and three-body descriptors of finite radial (10 Å) and angular (6 Å, *l*max = 3) cutoffs, with no explicit short-range repulsive wall; MACE is an equivariant message-passing neural network whose learned, higher-body-order representation and built-in short-range (ZBL) repulsion are pretrained across a broad, general data set. These features map directly onto the failure modes identified above: the missing repulsive wall that overcoordinates Li^+^, the limited angular resolution that rigidifies the Ca^2+^ shell, and the finite locality that undercaps the cooperative Cs^+^ cage are precisely the deficiencies a deeper, many-body, pretrained model is better placed to remedy. It is for this reason that MACE, although applied off-the-shelf without system-specific training, more faithfully tracks AIMD across both the structural and kinetic observables examined here.

### 3.4 Metadynamics Simulations

Metadynamics-derived free-energy profiles for the Mg^2+^ coordination number in aqueous MgCl_2_, computed using AMBER, the OT-MLFF, and a simple Lennard-Jones potential with ε = 0.1 kcal/mol, σ = 2.5 Å, and a cutoff of 10.0 Å, are presented in Figure S1. All three approaches locate a global free-energy minimum at CN ≈6, consistent with the established octahedral [Mg­(H_2_O)_6_]^2+^ complex, confirming that the dominant equilibrium coordination state is robust across force-evaluation strategies.

Marked differences are observed in the barrier topology. The AMBER profile exhibits steep, well-separated barriers between coordination states, reflecting the high energetic cost of ligand exchange for this strongly hydrated divalent cation. The OT-MLFF profile reproduces the CN = 6 minimum position but displays flattened plateaus and substantially reduced barrier slopes between coordination states, indicating a systematic underestimation of the activation free energies governing the first-shell exchange. These differences are directly reflected in the exchange dynamics of Table 2, in which the OT-MLFF generates 14 first-shell exchange events over 50 ps for Mg^2+^, a process absent from the AIMD trajectory and experimentally microsecond scale. The free-energy surface is converged with respect to the accumulated bias (Figure S2).

The barrier flattening in the OT-MLFF is most plausibly attributed to a training data coverage problem rather than a fundamental architectural limitation. Since Mg^2+^ first-shell exchange does not occur within the 50 ps NPT training trajectory at 300 K, the OT-MLFF is never presented with DFT reference forces in the transition-state region of the CN coordinate. The resulting potential energy surface in this region is an unvalidated interpolation that defaults to a smooth low-gradient profile. Augmenting the training set with configurations sampled by enhanced sampling simulations along the CN collective variable represents the most direct route to recovering barrier fidelity for strongly hydrated ions. For Mg^2+^, then, the OT-MLFF locates the correct equilibrium minimum but not the barriers around it (Section 3.2), pointing to transition-state sampling as the necessary remedy for predictive kinetics. The free-energy profiles are converged with respect to the accumulated bias (Figure S2).

### 3.5 Hydrogen-Bond Statistics

#### 3.5.1 Distribution of Hydrogen Bonds in Bulk Solution

Table 3 presents the distribution of hydrogen bonds (HBs) per water molecule obtained from AIMD, OT-MLFF, and MACE simulations. In pure water, the hydrogen-bond network is highly structured with most water molecules forming four hydrogen bonds. AIMD predicts a higher proportion of fully coordinated molecules (f_4_ = 70.7%) and a larger average HB number (3.75) compared to the OT-MLFF (59.9%, average 3.57), indicating a slightly stronger hydrogen-bond network in the AIMD description.

**Table 3:** Distribution of the Number of Hydrogen Bonds (HBs) per Water Molecule in Pure Water and LiCl, NaCl, KCl, CsCl, MgCl_2_, and CaCl_2_ Obtained from AIMD (DFT-PBE-D3) and MLFF Simulations of Hydrated Ions and Electrolyte Solution^a^

			Number of HBs (%) in bulk						
System	Method	b	f_0_	f_1_	f_2_	f_3_	f_4_	f_5_	Average
LiCl	OT-MLFF	0.9	0.5	4.9	9.4	33.9	48.2	2.9	3.34
LiCl	AIMD	0.9	0.1	0.8	7.0	26.1	62.6	3.4	3.61
LiCl	MACE	0.9	0.0	0.3	2.8	22.3	72.0	2.6	3.74
NaCl	OT-MLFF	0.9	0.1	1.3	10.4	32.8	50.2	5.3	3.48
NaCl	AIMD	0.9	0.1	1.2	8.3	27.9	58.8	3.7	3.55
NaCl	MACE	0.9	0.0	0.2	1.7	22.9	73.8	1.3	3.74
KCl	OT-MLFF	0.9	1.9	7.1	23.8	33.2	32.1	1.8	2.92
KCl	AIMD	0.9	0.1	1.0	7.0	25.6	62.5	3.8	3.61
KCl	MACE	0.9	0.0	0.3	5.2	23.8	67.6	3.2	3.68
CsCl	OT-MLFF	0.9	0.2	2.5	12.2	31.8	49.8	3.5	3.39
CsCl	AIMD	0.9	0.1	0.7	6.1	26.0	64.0	3.2	3.63
CsCl	MACE	0.9	0.0	0.2	2.9	20.4	73.9	2.6	3.76
MgCl_2_	OT-MLFF	0.9	0.2	3.5	15.3	33.9	44.8	2.3	3.27
MgCl_2_	AIMD	0.9	0.1	2.9	13.5	26.7	53.8	2.9	3.40
MgCl_2_	MACE	0.9	0.0	3.0	8.9	22.2	63.7	2.2	3.53
CaCl_2_	OT-MLFF	0.9	0.9	12.1	26.5	57.9	2.5	0.0	3.49
CaCl_2_	AIMD	0.9	0.1	2.9	12.3	25.2	57.3	2.3	3.43
CaCl_2_	MACE	0.9	0.01	0.7	9.1	27.9	59.3	2.6	3.51
H_2_O	OT-MLFF	–	0.2	1.6	8.7	25.2	59.9	4.4	3.57
H_2_O	AIMD	–	0.0	0.3	4.6	19.8	70.7	4.6	3.75
H_2_O	MACE	–	1.5	0.4	2.9	16.8	75.9	2.6	3.73

a The values are percentages of H_2_O with the given number of HBs. Concentration (b) in mol kg^–1^.

The hydrogen-bond network is naturally disrupted on addition of salts, leading to a shift toward lower coordination numbers (f_2_ and f_3_) and a reduction in the average number of hydrogen bonds. Among the monovalent salts, NaCl and CsCl show relatively minor perturbations, with average HB values close to pure water (≈3.4–3.55). In contrast, KCl MLFF shows a significantly reduced average HB number (2.92), indicating stronger disruption of the hydrogen-bond structure, although this effect is not reproduced as strongly in the AIMD results.

Divalent salts exhibit a more pronounced influence on the hydrogen-bond network upon switching to the MLFF. MgCl_2_ and CaCl_2_ solutions show larger populations of water molecules with two or three hydrogen bonds and correspondingly lower average HB values in AIMD (≈3.0). This behavior reflects the strong electrostatic interactions between highly charged cations and surrounding water molecules, which reorganize the HB structure and compete with water–water hydrogen bonding.

Overall, the OT-MLFF predicts a lower fraction of 4-fold-coordinated water molecules and smaller average HB numbers than AIMD in every system, indicating that it underestimates the stability of the hydrogen-bond network. MACE deviates in the opposite direction, overpredicting f_4_ in all six electrolytes while matching the AIMD description of pure water almost exactly (f_4_ = 75.9% vs 70.7%; average 3.73 vs 3.75 HBs per molecule). The two machine-learned potentials therefore bracket the AIMD reference, with the OT-MLFF understructuring and MACE overstructuring the network in the presence of ions. Nevertheless, all three methods capture the general trend of hydrogen-bond disruption upon salt addition, particularly for divalent electrolytes.

#### 3.5.2 Hydrogen-Bond Statistics in the Ionic Solvation Shell

Figure 6 shows the distribution of HBs per water molecule in the first solvation shell (FSS) of the ions obtained from AIMD, OT-MLFF, and MACE MLFF simulations. In the FSS of monovalent ions (Li^+^, Na^+^, K^+^, and Cs^+^), all three methods predict that water molecules predominantly form three to four hydrogen bonds, indicating that the hydrogen-bond network is only moderately perturbed. However, MLFF generally shifts the distribution toward lower HB numbers, particularly increasing the populations of two- and three-coordinated water molecules relative to AIMD. This effect becomes more pronounced for larger ions, such as K^+^ and Cs^+^. For Mg^2+^ and Ca^2+^, a stronger disruption of the HB network is observed with a larger fraction of water molecules exhibiting two to three HBs.

**Figure 6:**
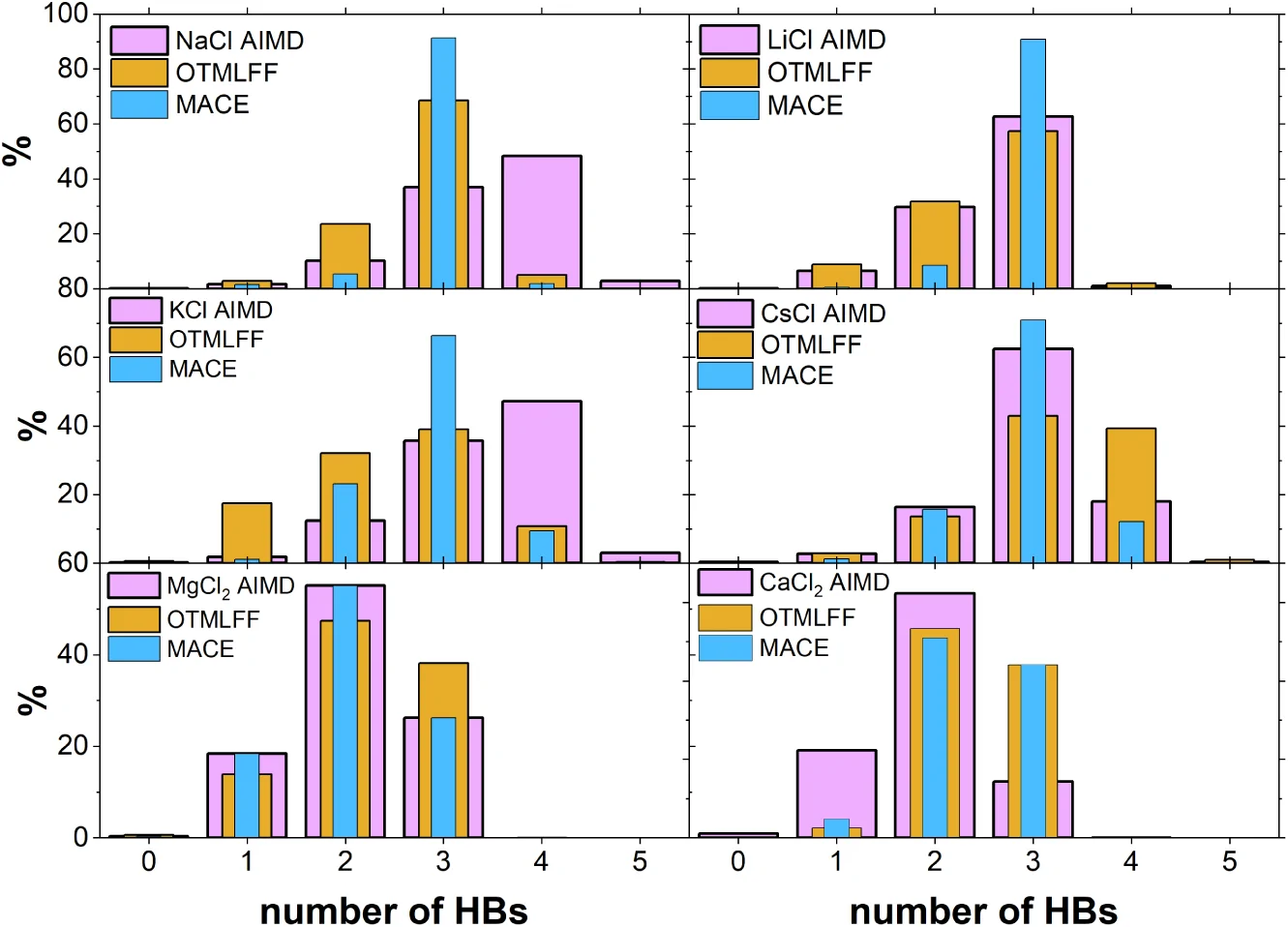
Percentage of water molecules that engage in *n* hydrogen bonds (HBs) in the first solvation shell for pure water and electrolyte solutions. Values obtained from AIMD (PBE-D3) and OT-MLFF and MACE trajectories.

Figure 7 presents the HB distribution for pure bulk water. AIMD (PBE-D3) predicts a strongly tetrahedral network, with 70.7% of water molecules forming four hydrogen bonds (f_4_) and an average of 3.75 HBs per molecule (Table 3). The OT-MLFF reproduces the qualitative shape of this distribution but systematically underestimates f_4_ (59.9%), redistributing population primarily into the three-coordinated state (f_3_: 25.2% vs 19.8% in AIMD), with minor elevations in f_2_. The average HB number from the OT-MLFF (3.57) is consequently lower than the AIMD reference by 0.18 HBs per molecule. This broadening of the HB distribution, a shift away from the fully tetrahedral four-coordinated state toward three-coordinated configurations, mirrors the angular smoothing that the OT-MLFF exhibits in the ion–water angular distribution functions (Figure 4): the same finite angular resolution that suppresses the O–Na–O shoulder near 65–70° and the cusps of the more labile shells is expected to to underestimate the energetic penalty associated with distortions of the water–water hydrogen-bond network, lowering the energetic cost of departing from tetrahedral geometry relative to the PBE-D3 reference and redistributing population from four- to three-coordinated states. We note that the magnitude of this shift (f_4_ = 70.7% → 59.9%; average 3.75 → 3.57) is comparable to the difference reported between GGA and meta-GGA descriptions of liquid water (f_4_ ≈72% → 56% and average ≈3.77 → 3.61 between PBE and SCAN^38^), indicating that the hydrogen-bond populations are a sensitive diagnostic of the underlying angular potential rather than a marginal artifact of the OT-MLFF. Notably, all three methods agree that f_0_ is negligible and that f_5_ is a minor population (∼4.4–4.6%), confirming that fully uncoordinated and overcoordinated water molecules are rare under ambient conditions regardless of the force evaluation method.

**Figure 7:**
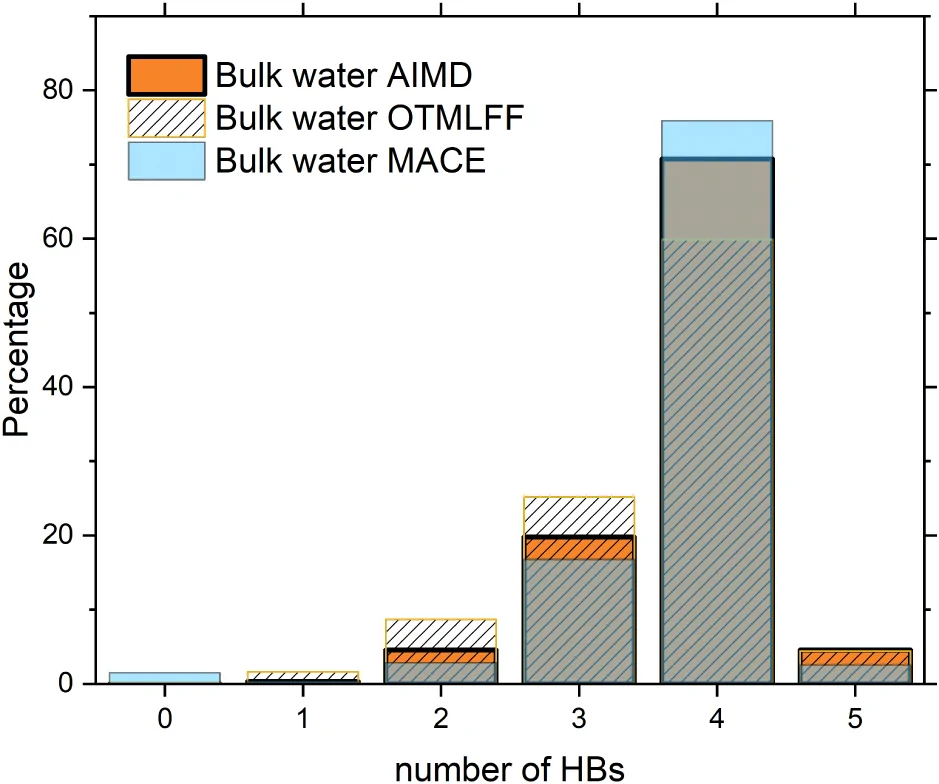
Percentage of water molecules that engage in *n* hydrogen bonds (HBs) in pure water. Values obtained from AIMD (PBE-D3) and OT-MLFF and MACE trajectories.

## 4 Conclusions

This study presents a systematic benchmarking of the on-the-fly machine learning force field (OT-MLFF) framework implemented in VASP against AIMD and classical CMD for aqueous solutions of six ions spanning a wide range of charge densities and hydration time scales: the monovalent alkali cations Li^+^, Na^+^, K^+^, and Cs^+^ and the divalent alkaline-earth cations Mg^2+^ and Ca^2+^. By evaluating structural, dynamical, and thermodynamic observables within a single, consistent computational framework, this work provides the first comprehensive assessment of the OT-MLFF approach, which generates its training data autonomously during simulation via Bayesian force error estimation, for liquid-phase ion solvation across a chemically diverse cation series.

The central finding of this benchmark is a systematic dissociation between structural and dynamical fidelity in the OT-MLFF. Equilibrium solvation structural properties such as bulk-water RDFs, the M–O distance trend down both series, and the coordination-number distributions of Na^+^, K^+^, and both divalent ions are reproduced with near-AIMD accuracy, with the single structural exception of a mild Li^+^ (and Cs^+^) overcoordination. Dynamical and kinetic observables, by contrast, are reproduced far less reliably: the OT-MLFF generates spurious first-shell exchange for Mg^2+^ (14 events in 50 ps against none in AIMD), completely suppresses the dynamic Ca^2+^ shell (zero events against 10 in AIMD), and qualitatively misrepresents the cooperative cage dynamics of the large monovalent ions Cs^+^ and K^+^. The Ca^2+^ case is the sharpest illustration of this dissociation: at the 1 mol dm^–3^ composition studied here, all three methods agree on a six-coordinate shell, yet only AIMD and MACE reproduce its exchange kinetics, so the OT-MLFF reproduces the *structure* of the Ca^2+^ shell while failing to reproduce its *dynamics*. Thermodynamic accuracy at the level of the free-energy minimum therefore does not imply kinetic accuracy at the level of the barriers between coordination states, a distinction of direct practical consequence for any application in which the OT-MLFF is used to predict rates, residence times, or transport rather than static structure.

### 4.1 Where the OT-MLFF Succeeds

For equilibrium structural observables, the OT-MLFF consistently outperforms CMD and approaches AIMD accuracy across the ion series: it reproduces the bulk-water O–O and O–H RDFs, the systematic M–O distance trend down both series, and the coordination-number distributions of Na^+^, K^+^, Cs^+^, and both divalent ions (Sections 3.1.1–3.1.3), together with the rigid octahedral Mg^2+^ geometry in both its CN and O–Mg–O angular distribution. Short-time cage dynamics are captured for Li^+^, Mg^2+^, and Ca^2+^ and first-shell exchange kinetics for Na^+^ and K^+^ (Sections 3.2–3.3). One structural property is reproduced only moderately: the OT-MLFF underestimates the tetrahedral hydrogen-bond population of bulk water (Section 3.5), so its radial structure is more faithful than its angular hydrogen-bond network. MACE reproduces the same equilibrium structure with comparable fidelity and additionally recovers the divalent exchange kinetics that the OT-MLFF misses.

### 4.2 Where the OT-MLFF Fails

Despite these successes, the OT-MLFF exhibits systematic and ion-specific failures that are consequential for predictive applications. These failures fall into three distinct categories. The first is coordination-number bias for edge-case ions. The OT-MLFF mildly overcoordinates Li^+^, which we attribute to an incomplete description of the steep short-range repulsion of this small, highly charged ion’s compact first shell. Ca^2+^, by contrast, is reproduced structurally by both machine-learned potentials at the 1 mol dm^–3^ composition studied here and is therefore no longer a structural failure of the OT-MLFF; its shortcoming for this ion is purely kinetic (below).

The second is three-body angular sensitivity. The O–M–O angular distribution functions show that the OT-MLFF smooths subtle but physically meaningful features of the three-body potential energy surface for ions with labile first shells. For Ca^2+^, this angular stiffening does not coincide with a coordination-number error but instead manifests kinetically, suppressing the fluctuations required for exchange, whereas the higher angular resolution of MACE better reproduces the flexible Ca^2+^ landscape. We attribute these angular errors to the difficulty of representing the nonlinear, many-body angular potential within a kernel-based descriptor of finite angular resolution.

The third is the underestimation of kinetic barriers. For Mg^2+^, the OT-MLFF generates spurious first-shell exchange absent in AIMD, and for Ca^2+^, it suppresses the exchange that AIMD exhibits; the Mg^2+^ metadynamics profiles confirm that the OT-MLFF preserves the equilibrium minimum but flattens the barriers between coordination states. MACE removes both kinetic failures, indicating that the overflat barrier topology is specific to the kernel OT-MLFF rather than intrinsic to machine-learned potentials. These failures show that thermodynamic fidelity does not guarantee kinetic fidelity: the OT-MLFF potential energy surface is insufficiently steep for highly charged or borderline hard cations.

Finally, the velocity autocorrelation function analysis reveals an additional failure mode not apparent from equilibrium structural observables: the OT-MLFF fundamentally misrepresents the caging dynamics of Cs^+^, predicting a shallow near-zero backscattering minimum in contrast to the deep minimum of approximately −0.4 at ∼500 fs observed in AIMD. A qualitatively similar but less severe discrepancy is observed for K^+^. These failures are consistent with the collective, cooperative nature of the cage imposed by large, high-CN hydration shells, which may exceed what the finite OT-MLFF cutoff can capture.

### 4.3 Origin of Failures and Recommended Improvements

The pattern of OT-MLFF successes and failures across structural, angular, kinetic, and dynamical observables points to three underlying limitations of the current implementation. First, the locality approximation, decomposition of the total energy into a sum of atomic contributions within a finite radial cutoff, is insufficient for capturing the cooperative many-body interactions that govern cage dynamics for large, weakly polarizing cations and the subtle angular energy landscape for borderline-hard divalent cations. Second, the angular descriptor resolution (*l*_max_ = 3, angular cutoff 6 Å) is insufficient to represent the nonlinear, many-body character of the three-body potential energy surface for ions such as Ca^2+^, whose coordination geometry lies between the well-defined octahedral limit of Mg^2+^ and the fully disordered limit of Cs^+^. Third, the on-the-fly training protocol, while efficient, may undersample the transition-state configurations, transient over- and undercoordinated geometries, that are critical for accurately representing exchange kinetics but are visited infrequently during equilibrium MD trajectories. These are, notably, the limitations that the equivariant, pretrained MACE model is architecturally equipped to address: its higher body order and learned representation supply the angular flexibility, its built-in short-range repulsion supplies the missing Li^+^ wall, and its broad pretraining reduces reliance on system-specific transition-state sampling. The systematic improvement of MACE over the OT-MLFF across the structural and kinetic observables reported here is consistent with this attribution. That these gains are achieved by MACE-MP-0 *off-the-shelf*, without any fine-tuning on the present systems, indicates that the improvements stem from the architecture and broad pretraining rather than system-specific adaptation; fine-tuning represents an available but, for the Li^+^ and Ca^2+^ discrepancies highlighted here, unnecessary further step.

Targeted improvements to address these limitations include the incorporation of long-range electrostatic corrections, such as charge equilibration schemes or the addition of a physics-based long-range tail to the kernel-based potential, to improve cooperative cage dynamics for large monovalent cations; the use of higher angular resolution descriptors or equivariant architectures (e.g., MACE-class models) to improve three-body sensitivity for Ca^2+^-like systems; and the augmentation of the on-the-fly training data set with configurations drawn from enhanced sampling simulations, metadynamics, or umbrella sampling along the coordination-number collective variable, to improve the representation of exchange transition states and thereby recover kinetic fidelity without sacrificing the efficiency of the automated OT-MLFF workflow. The present results support this: a general-purpose MACE potential, applied without system-specific training, reproduces several kinetic features that the on-the-fly MLFF fails to capture.

We note that these mechanistic attributions, while consistent across the structural, angular, and kinetic observables and reinforced by the contrasting behavior of the equivariant MACE model, are interpretations rather than uniquely determined conclusions: the present data establish *where* the OT-MLFF fails with high confidence, but disentangling the relative contributions of locality, angular-descriptor resolution, and training-data coverage would require controlled ablations (e.g., systematic variation of the cutoff and *l*_max_) that lie beyond the scope of this benchmark.

### 4.4 Broader Implications

The results presented here establish that the OT-MLFF framework efficiently reproduces the equilibrium pair structure of aqueous electrolyte solutions across a chemically diverse ion series, at a fraction of the computational cost of AIMD, but that its reliability decreases systematically as the target observable becomes more sensitive to three-body angular topology and exchange barrier kinetics. It is thus a dependable tool for the first-shell solvation structure, while dynamical and angular observables such as hydrogen-bond tetrahedrality, Cs^+^/K^+^ caging, and divalent exchange kinetics require the higher body order of a pretrained equivariant model such as MACE, which recovers several of these properties that the OT-MLFF does not. The identification of its specific limitations and their mechanistic attribution to the locality approximation, angular descriptor resolution, and training data coverage provide a clear roadmap for targeted improvements. As electrolyte modeling increasingly underpins the design of next-generation battery materials, desalination membranes, and biomineralization processes, the development of OT-MLFF protocols that combine the accessibility of automated training with the accuracy of equivariant or long-range-corrected architectures represents a high-priority direction for the field. The complementary strengths identified here, the autonomous, system-specific training of the OT-MLFF and the transferable, higher-body-order accuracy of a pretrained model such as MACE, suggest that hybrid protocols combining on-the-fly data generation with equivariant, long-range-corrected architectures are a particularly promising route.

## Supplementary Material



## Notes

Composition, cell dimensions and trajectory length of every simulated system (Table S1); structural parameters of the AIMD cation–water radial distribution functions compared with previous ab initio studies and experiment (Table S2); metadynamics free-energy profiles for the Mg^2+^ coordination number (Figure S1); convergence of the well-tempered metadynamics free-energy surface (Figure S2); out-of-sample validation of the OT-MLFF against single-point PBE-D3 calculations, force and energy parity plots, and per-system error statistics (Section S3, Table S3, Figure S3); and comparison of AIMD radial distribution functions computed with VASP and with CP2K (Figure S4) (PDF)
