# Supplementary material for: Benchmarking on-the-Fly Machine Learning Force Fields for Ion Hydration: Structure, Coordination, and Exchange Dynamics across Monovalent and Divalent Cations
Source: J Phys Chem B. 2026 Sep 2;130(37):9543–60. doi: 10.1021/acs.jpcb.6c02837 (PMC13592074; doi:10.1021/acs.jpcb.6c02837)
Supplement: Supplementary file 1 [file jp6c02837_si_001.pdf]

Supplementary Material for "**Benchmarking On-the-Fly Machine Learning Force Fields for Ion Hydration: Structure, Coordination, and Exchange Dynamics across Monovalent and Divalent Cations**"

Mohammed S. Salha[a], Wuyang Lin[a], Greg Chass[a], and Devis Di Tommaso[a,*]

[a] Department of Chemistry, School of Physical and Chemical Sciences, Queen Mary University of London, Mile End Road, London, E1 4NS, United Kingdom

* Corresponding author. E-mail address: d.ditommaso@qmul.ac.uk (D. Di Tommaso)

TABLE S1. Details of the electrolyte solutions: concentration (b) in mol.kg^{-1}, number of units and H_2O molecules, cell length after classical MD (NPT) simulation, and total time duration (t_{sim}) of each AIMD simulation. All six salts and bulk water were simulated at the same concentration (0.9 mol kg–1; a single ion pair in ~62 H2O) with the OT-MLFF, AIMD, and MACE methods; the MACE trajectories used the same simulation cells as the corresponding AIMD boxes.

System	Method	b	n_{units}	n_{water}	Cell length (Å)	t_{sim}
LiCl-MLFF	MLFF	0.9	1	62	11.9	50
LiCl-AIMD	AIMD	0.9	1	62	12.3	50
LiCl-MACE	MACE	0.9	1	62	12.3	50
NaCl-MLFF	MLFF	0.9	1	62	12.5	50
NaCl-AIMD	AIMD	0.9	1	62	12.3	50
NaCl-MACE	MACE	0.9	1	62	12.3	50
KCl-MLFF	MLFF	0.9	1	62	12.6	50
KCl-AIMD	AIMD	0.9	1	62	12.4	50
KCl-MACE	MACE	0.9	1	62	12.4	50
CsCl-MLFF	MLFF	0.9	1	62	12.6	50
CsCl-AIMD	AIMD	0.9	1	62	12.6	50
CsCl-MACE	MACE	0.9	1	62	12.6	50
MgCl2-MLFF	MLFF	0.9	1	62	12.3	50
MgCl2-AIMD	AIMD	0.9	1	62	12.3	50
MgCl2-MACE	MACE	0.9	1	62	12.3	50
CaCl2-MLFF	MLFF	0.9	1	62	12.1	50
CaCl2-AIMD	AIMD	0.9	1	62	12.4	50
CaCl2-MACE	MACE	0.9	1	62	12.4	50
Bulk water-MLFF	MLFF	0.0	–	64	12.4	50
Bulk water-AIMD	AIMD	0.0	–	64	12.5	50
Bulk water-MACE	MACE	0.0	–	64	12.4	50

TABLE S2. Structural properties of the cation–water radial distribution functions obtained from the AIMD simulations of the hydrated ions (isolated ion, no counterion) in 63 water molecules conducted in this study. The positions, r_{max}, and amplitudes, $g(r_{max})$, of first peak and the average coordination number (CN) of the cation hydration shell are compared with other AIMD studies and available experimental data. Distances in Å.

System	Reference	Method [a)]	Functional	Pseudopot. [b)]	Basis set [c)]	r^{max}	$g(r^{max})$	CN
Li^{+}	This study	BOMD	PBE-D3	GTH	DZVP/PW (1000 Ry)	1.98	9.4	4.0
	MLFF	This study	PBE-D3	PAW	PW (500 eV)	1.95	10.2	4.97
	Lyubartsev[1]	CPMD	BLYP	USPP	PW (25 Ry)	1.96	9.5	
Na^{+}	This study	BOMD	PBE-D3	GTH	DZVP/PW (1000 Ry)	2.41	5.0	5.1
	This study	MLFF	PBE-D3	PAW	PW (500 eV)	2.34	6.4	5.00
		BOMD	revPBE-D3	GTH	DZVP/PW (1000 Ry)	2.53	4.6	5.6
	Duignan et al.[2]	BOMD	revPBE-D3	GTH	DZVP/PW (400 Ry)	2.51	4.1	
		BOMD	SCAN	GTH	DZVP/PW (1200 Ry)	2.36	5.9	
	Galib et al.[3]	BOMD	revPBE	GTH	TZV2P/PW (400 Ry)	2.45	5.8	5.7
		BOMD	revPBE-D3	GTH	TZV2P/PW (400 Ry)	2.53	4.8	6.0
		BOMD	revPBE	GTH	DZVP/PW (400 Ry)	2.46	5.7	5.7
		BOMD	revPBE-D3	GTH	DZVP/PW (400 Ry)	2.56	4.0	6.1
		BOMD	BLYP	GTH	TZV2P/PW (400 Ry)	2.40	5.9	4.9
		BOMD	BLYP-D2	GTH	TZV2P/PW (400 Ry)	2.46	6.4	5.7
NaCl (6 m)	Galib et al.[3]	EXAFS				2.37		5.4
		XRD				2.38		5.5
NaCl (2.5 m)	Galib et al.[3]	XRD				2.38		5.9
K^{+}	This study	BOMD	PBE-D3	GTH	DZVP/PW (1000 Ry)	2.80	3.4	6.2
	This study	MLFF	PBE-D3	PAW	PW (500 eV)	2.79	4.2	5.62
	Duignan et al.[2]	BOMD	revPBE-D3	GTH	DZVP/PW (400 Ry)	2.98	3.1	
		BOMD	SCAN	GTH	DZVP/PW (1200 Ry)	2.78	3.8	
	Glezakou et al.[4]	EXAFS				2.76		6.1
Cs^{+}	This study	BOMD	PBE-D3	GTH	DZVP/PW (1000 Ry)	3.17	2.7	5.9
	This study	MLFF	PBE-D3	PAW	PW (500 eV)	3.05	3.6	4.38
	Roy et al.[5]	BOMD	PBE-D3	PAW	PW (30 Ry)	3.15	3.3	

System	Reference	Method [a)]	Functional	Pseudopot. [b)]	Basis set [c)]	r^{max}	$g(r^{max})$	CN
Mg^{2+}	This study	BOMD	PBE-D3	GTH	DZVP/PW (1000 Ry)	2.11	13.5	6.0
	This study	MLFF	PBE-D3	PAW	PW (500 eV)	2.08	19.7	5.97
	Di Tommaso[6]	CPMD	PBE	USPP	PW (30 Ry)	2.08	12.3	6.0
	Callahan et al.[7]	XRD				2.0–2.12		6.0
Ca^{2+}	This study	BOMD	PBE	GTH	DZVP/PW (1000 Ry)	2.40	10.8	6.0
		BOMD	PBE-D3	GTH	DZVP/PW (1000 Ry)	2.40	10.2	6.0
		BOMD	BLYP	GTH	DZVP/PW (1000 Ry)	2.40	9.6	6.3
		BOMD	BLYP-D3	GTH	DZVP/PW (1000 Ry)	2.42	9.9	6.8
		BOMD	revPBE	GTH	DZVP/PW (1000 Ry)	2.43	9.4	6.6
		BOMD	revPBE-D3	GTH	DZVP/PW (1000 Ry)	2.39	10.8	6.1
	This study	MLFF	PBE-D3	PAW	PW (500 eV)	2.34	11.0	6.00
	Di Tommaso et al.[6]	CPMD	PBE-D3	GTH	DZVP/PW (1000 Ry)	2.36	8.5	6.4
	Bako et al.[8]	CPMD	BLYP	NCPP	PW (70 Ry)	2.45	10.5	6
$CaCl_2$ (6 m)	Fulton et al.[9]					2.43		7.2

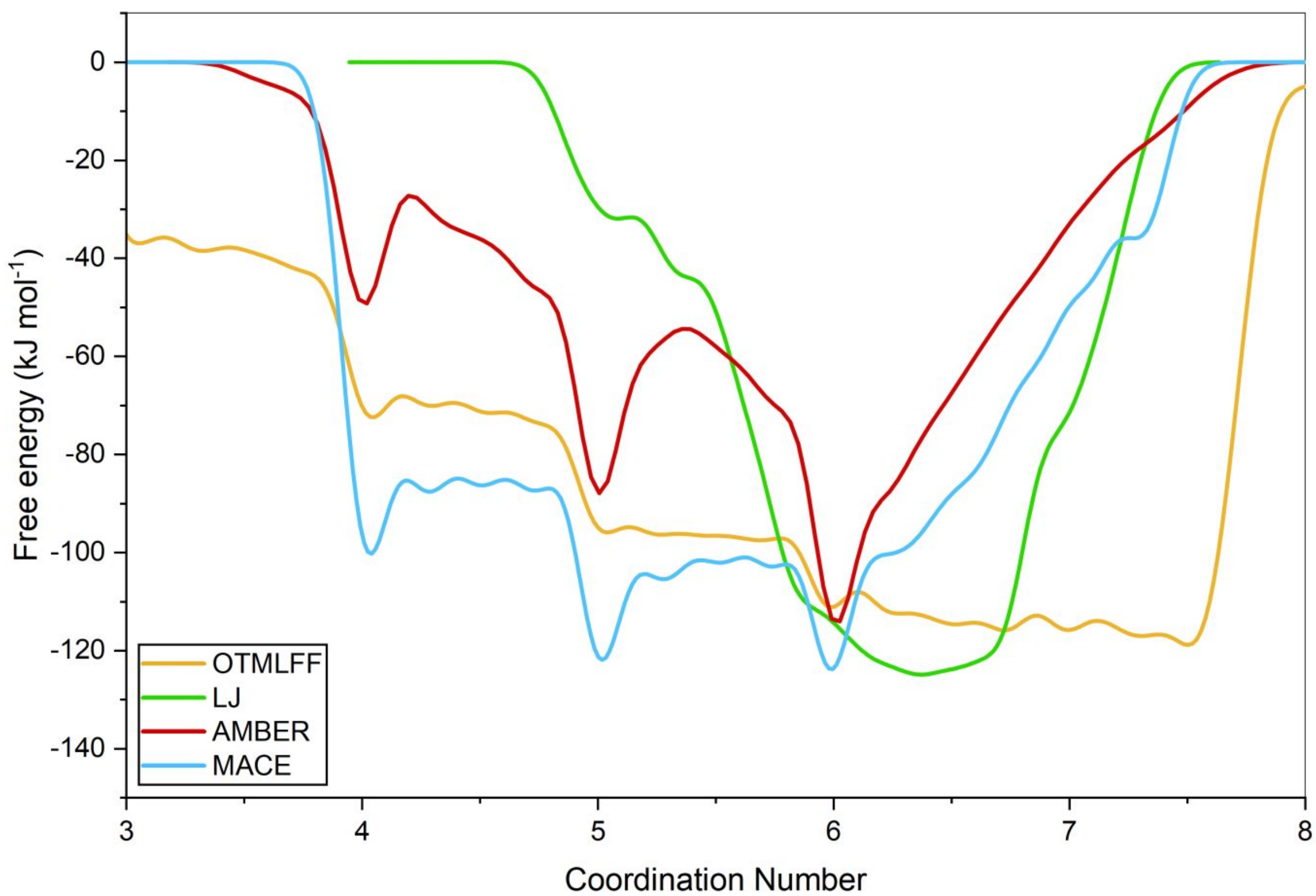

FIGURE S1. Free energy as a function of the ion–water coordination number computed from well-tempered metadynamics-biased MD method. Minima deviate slightly from the expected integer coordination values due to numerical factors arising from the need to define a smooth and continuous analytic definition of the coordination number for use as a collective variable.

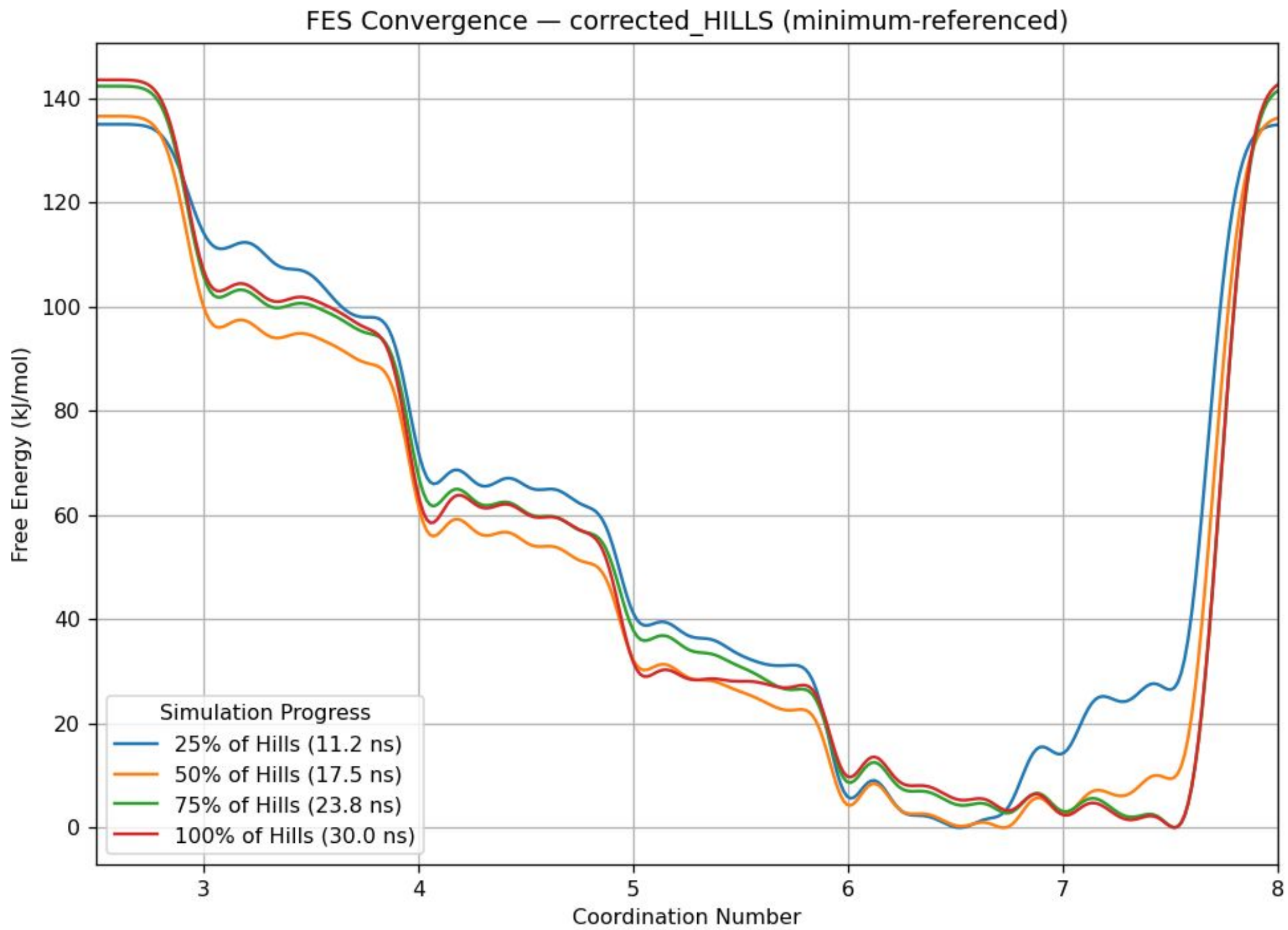

FIGURE S2. Convergence of the well-tempered metadynamics free-energy surface for the OT-MLFF Mg^{2+} CN. Free energy as a function of the Mg–O coordination number, reconstructed from the Gaussian hills accumulated over 25 %, 50 %, 75 % and 100 % of the 30 ns biased trajectory (11.2, 17.5, 23.8 and 30.0 ns, respectively). Metadynamics parameters: bias factor $\gamma = 5$, initial hill height 1.0 kJ mol^{-1}, width $\sigma = 0.1$, deposition stride 1000 steps, $T = 300$ K. The near-superposition of the 75 % and 100 % profiles indicates a converged surface.

S3. Out-of-sample validation of the OT-MLFF against DFT

To quantify the accuracy of the on-the-fly force fields on configurations not encountered during training, 200 statistically decorrelated frames were extracted at regular intervals from the final 90% of each production OT-MLFF trajectory. Because the production runs were propagated in pure prediction mode (ML_MODE = run), no on-the-fly DFT calculations are triggered on these frames, so they constitute a genuine out-of-sample test set. Each frame was recomputed with a single-point DFT calculation at the OT-MLFF training level (PBE-D3, 500 eV plane-wave cutoff, PAW), and the OT-MLFF-predicted atomic forces and total energies were compared with the DFT reference. Force errors are the root-mean-square (RMSE) and mean-absolute (MAE) deviations over all Cartesian force components; R is the Pearson correlation of the force components; the energy RMSE is reported per atom after removing a constant offset between the two energy references.

Across the six electrolytes (Table S3, Figure S3) the OT-MLFF reproduces the DFT forces with an RMSE of 78–84 meV Å–1 (mean 81 meV Å–1) and a force-component correlation of R = 0.994 for every system, and the total energies to within 1.6–2.0 meV atom–1. The force and energy accuracy is essentially uniform across the ion series: the RMSE varies by less than 8% between the most and least accurate system and shows no systematic dependence on ionic charge or radius. This uniformity indicates that the ion-specific structural and dynamical discrepancies discussed in the main text (e.g. the Li+ over-coordination and the divalent water-exchange failures) do not arise from a locally poorer force fit for those ions, but reflect the model's response to the shape of the potential-energy surface. The force errors are, moreover, competitive with those reported for bespoke graph-neural-network interatomic potentials of aqueous and condensed-phase systems, which typically lie in the few-tens to ~100 meV Å–1 range.

TABLE S3. Out-of-sample validation of the OT-MLFF against the PBE-D3 DFT reference. For each electrolyte, 200 production frames were recomputed at the DFT level. Force RMSE and MAE are over all Cartesian components (meV Å–1); R is the force-component Pearson correlation; energy RMSE is per atom (meV atom–1) after removal of a constant offset.

**System**	**Frames**	**Force RMSE (meV Å–1)**	**Force MAE (meV Å–1)**	**R**	**Energy RMSE (meV atom–1)**
LiCl	200	80.7	59.4	0.9942	1.65
NaCl	200	81.0	60.0	0.9942	1.63
KCl	200	81.8	60.0	0.9942	1.81
CsCl	200	77.7	57.4	0.9947	1.64
MgCl2	200	79.6	58.3	0.9944	2.00
CaCl2	200	83.9	61.6	0.9938	1.67
**Mean**	**—**	**80.8**	**59.5**	**0.9942**	**1.73**

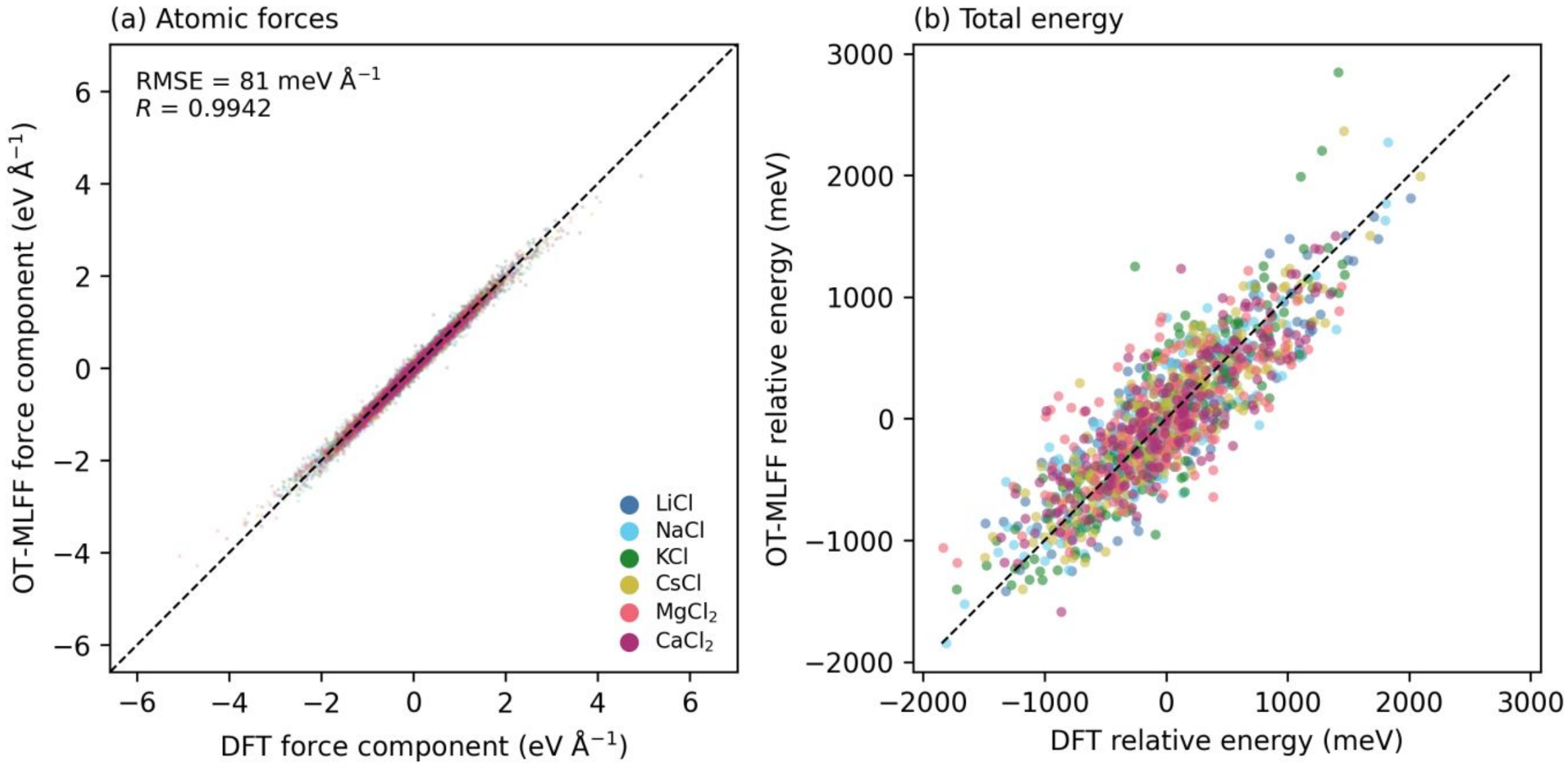

FIGURE S3. Parity between OT-MLFF-predicted and DFT-computed (a) atomic force components and (b) total energies (referenced to the per-system mean), for 200 out-of-sample production frames of each electrolyte (colours). The dashed line is the identity. Over the full set, force RMSE = 81 meV Å–1 and R = 0.994.

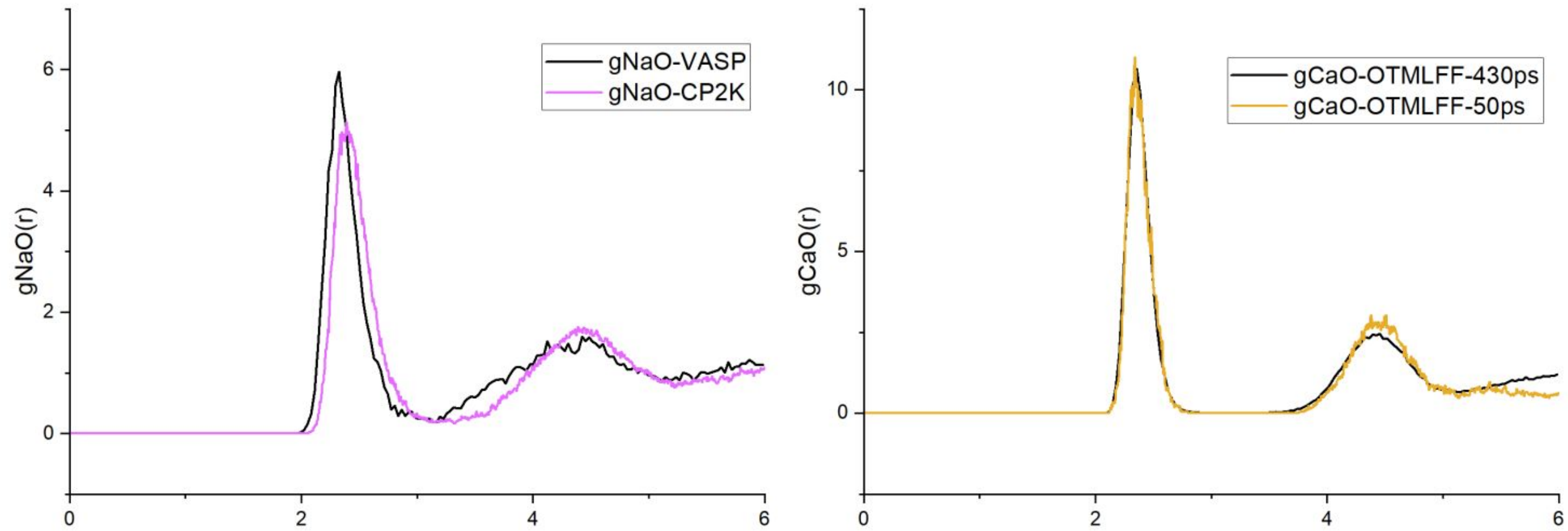

FIGURE S4. (a) Sodium–oxygen radial distribution function of aqueous NaCl from AIMD at the PBE-D3 level computed with VASP (black) and with CP2K (magenta), illustrating the effect of the basis and pseudopotential representation on peak intensity. (b) Calcium–oxygen radial distribution function of aqueous $CaCl_2$ from the OT-MLFF, computed over the 50 ps production trajectory (yellow) and over an extended 430 ps trajectory (black), confirming that the production window is sufficient for the equilibrium structural observables reported in the main text.
